# Supplementary material for: MicroRNAomic Analysis of Spent Media from Slow- and Fast-Growing Bovine Embryos Reveal Distinct Differences
Source: Animals (Basel). 2024 Aug 13;14(16):2331. doi: 10.3390/ani14162331 (PMC11350645; doi:10.3390/ani14162331)
Supplement: Supplementary file 1 [file animals-14-02331-s001.zip › animals-3022580-supplementary.pdf]

Supplementary Material

Table S1. Predicted mRNA targets of miRNAs differentially expressed between 2-cell SG

miRNA	Gene	Cumulative Context Score	miRNA	Gene	Cumulative Context Score
bta-miR-1343-5p	PRX	-1.51	bta-miR-2393	SRGAP3	-1
bta-miR-1343-5p	KIAA0513	-1.48	bta-miR-2393	CLMP	-1
bta-miR-1343-5p	KSR2	-1.29	bta-miR-2393	MECP2	-1
bta-miR-1343-5p	RAB37	-1.27	bta-miR-2393	NKX2-1	-1
bta-miR-1343-5p	LY6G6C	-1.24	bta-miR-2393	KLF12	-1
bta-miR-1343-5p	EHD2	-1.22	bta-miR-2393	NKD1	-1
bta-miR-1343-5p	C17orf103	-1.13	bta-miR-2393	ETV6	-1
bta-miR-1343-5p	GAS8	-1.1	bta-miR-2393	RORA	-1
bta-miR-1343-5p	RGMA	-1.03	bta-miR-2393	BRCA2	-0.85
bta-miR-1343-5p	OSM	-1	bta-miR-2393	RP11-67H2.1	-0.74
bta-miR-1343-5p	LYPD1	-0.95	bta-miR-2393	CYLC2	-0.69
bta-miR-1343-5p	PKD1	-0.95	bta-miR-2393	FAM171B	-0.63
bta-miR-1343-5p	MS4A15	-0.95	bta-miR-2393	C21orf37	-0.61
bta-miR-1343-5p	GPA33	-0.95	bta-miR-2393	TTC30B	-0.58
bta-miR-1343-5p	COTL1	-0.92	bta-miR-2393	DNAJC1	-0.55
bta-miR-1343-5p	FKBP1A	-0.91	bta-miR-2393	OR51I2	-0.55
bta-miR-1343-5p	HOXB5	-0.91	bta-miR-2393	GYPA	-0.54
bta-miR-1343-5p	SPSB1	-0.86	bta-miR-2393	SLC25A31	-0.54
bta-miR-1343-5p	TFCP2L1	-0.86	bta-miR-2393	KRCC1	-0.54
bta-miR-1343-5p	TFEB	-0.86	bta-miR-2393	FAM213A	-0.52
bta-miR-1343-5p	FHL3	-0.86	bta-miR-2393	AC090186.1	-0.51
bta-miR-1343-5p	SIPA1L3	-0.85	bta-miR-2393	OR4F4	-0.51
bta-miR-1343-5p	LDLRAP1	-0.84	bta-miR-2393	DYDC1	-0.5
bta-miR-1343-5p	KCTD17	-0.83	bta-miR-2393	PMPCB	-0.5
bta-miR-1343-5p	SIX5	-0.83	bta-miR-2412	PML	-1.17
bta-miR-1343-5p	KB-1507C5.2	-0.82	bta-miR-2412	COX6B2	-1.09
bta-miR-1343-5p	MVB12B	-0.82	bta-miR-2412	TNFSF13	-1.04
bta-miR-1343-5p	ATG9A	-0.8	bta-miR-2412	C15orf32	-1.03
bta-miR-1343-5p	HMGA1	-0.8	bta-miR-2412	WNT4	-0.97
bta-miR-1343-5p	TBC1D13	-0.79	bta-miR-2412	CTD-3203P2.2	-0.91
bta-miR-1343-5p	GRB7	-0.79	bta-miR-2412	SNX32	-0.85
bta-miR-1343-5p	KLK14	-0.78	bta-miR-2412	TNFSF12-TNFSF13	-0.85
bta-miR-1343-5p	UBE2QL1	-0.78	bta-miR-2412	SLC34A2	-0.81
bta-miR-1343-5p	THTPA	-0.77	bta-miR-2412	C1QTNF6	-0.78
bta-miR-1343-5p	PIRT	-0.77	bta-miR-2412	KXD1	-0.75
bta-miR-1343-5p	TNS4	-0.77	bta-miR-2412	CPLX3	-0.71
bta-miR-1343-5p	HEPACAM	-0.77	bta-miR-2412	RBPMS2	-0.68
bta-miR-1343-5p	STIM1	-0.77	bta-miR-2412	CRABP2	-0.66
bta-miR-1343-5p	RRP1	-0.76	bta-miR-2412	RFX1	-0.65
bta-miR-1343-5p	PACRG	-0.76	bta-miR-2412	SPATA3	-0.59
bta-miR-1343-5p	PSORS1C1	-0.76	bta-miR-2412	FAM53B	-0.59

bta-miR-1343-5p	GAREML	-0.76	bta-miR-2412	GRAP	-0.58
bta-miR-1343-5p	NUPR1	-0.75	bta-miR-2412	PSCA	-0.58
bta-miR-1343-5p	SERPINE3	-0.75	bta-miR-2412	CMIP	-0.58
bta-miR-1343-5p	AQP5	-0.75	bta-miR-2412	FAM98C	-0.57
bta-miR-1343-5p	TSTA3	-0.75	bta-miR-2412	PIP5K1C	-0.57
bta-miR-1343-5p	GLIS1	-0.75	bta-miR-2412	SEPT5	-0.56
bta-miR-1343-5p	SEC14L6	-0.74	bta-miR-2412	LZTS1	-0.56
bta-miR-1343-5p	LY6E	-0.74	bta-miR-2412	SUV39H1	-0.56
bta-miR-1343-5p	CAMKV	-0.74	bta-miR-2412	FAM102A	-0.55
bta-miR-1343-5p	TMEM150A	-0.73	bta-miR-2412	IL18RAP	-0.54
bta-miR-1343-5p	TMCC2	-0.73	bta-miR-2412	PRPH	-0.54
bta-miR-1343-5p	SCAND1	-0.73	bta-miR-2412	ENPP7	-0.53
bta-miR-1343-5p	LOH12CR1	-0.72	bta-miR-2412	TARBP2	-0.52
bta-miR-1343-5p	IL17REL	-0.72	bta-miR-2412	GBP5	-0.52
bta-miR-1343-5p	TMEM27	-0.71	bta-miR-2412	AMZ1	-0.51
bta-miR-1343-5p	PRRT2	-0.71	bta-miR-2412	GBGT1	-0.51
bta-miR-1343-5p	ABHD14B	-0.71	bta-miR-2412	ZNRF3	-0.5
bta-miR-1343-5p	RNASEH2A	-0.71	bta-miR-2421	XKR4	-2.17
bta-miR-1343-5p	JPH4	-0.71	bta-miR-2421	NFIA	-1.79
bta-miR-1343-5p	NDRG4	-0.71	bta-miR-2421	ONECUT2	-1.5
bta-miR-1343-5p	RARG	-0.7	bta-miR-2421	TCF4	-1.21
bta-miR-1343-5p	LIF	-0.7	bta-miR-2421	ELAVL4	-1.14
bta-miR-1343-5p	CABP7	-0.7	bta-miR-2421	NFIB	-1.09
bta-miR-1343-5p	RIMS4	-0.7	bta-miR-2421	GABRB3	-1
bta-miR-1343-5p	AIF1L	-0.7	bta-miR-2421	THRB	-1
bta-miR-1343-5p	PTMS	-0.7	bta-miR-2421	POU6F2	-0.99
bta-miR-1343-5p	CASQ1	-0.7	bta-miR-2421	TNRC6C	-0.92
bta-miR-1343-5p	SERPINB6	-0.7	bta-miR-2421	IGIP	-0.91
bta-miR-1343-5p	GJB4	-0.7	bta-miR-2421	TNRC6B	-0.87
bta-miR-1343-5p	KIAA1644	-0.7	bta-miR-2421	GLIPR1L1	-0.82
bta-miR-1343-5p	FAM178B	-0.69	bta-miR-2421	CYLC2	-0.81
bta-miR-1343-5p	GPIHBP1	-0.69	bta-miR-2421	RUNX1T1	-0.75
bta-miR-1343-5p	BLOC1S3	-0.68	bta-miR-2421	PRR26	-0.74
bta-miR-1343-5p	FAM222B	-0.68	bta-miR-2421	ZFHX3	-0.71
bta-miR-1343-5p	SLC6A8	-0.68	bta-miR-2421	TSHZ2	-0.7
bta-miR-1343-5p	USP21	-0.68	bta-miR-2421	NECAB1	-0.7
bta-miR-1343-5p	ZNF703	-0.68	bta-miR-2421	ELAVL3	-0.65
bta-miR-1343-5p	VAMP2	-0.67	bta-miR-2421	AL359878.1	-0.6
bta-miR-1343-5p	ASTN1	-0.67	bta-miR-2421	LOH12CR2	-0.54
bta-miR-1343-5p	FCRL6	-0.67	bta-miR-2421	RBMS1	-0.54
bta-miR-1343-5p	APLNR	-0.67	bta-miR-2421	PCLO	-0.54
bta-miR-1343-5p	DDX41	-0.67	bta-miR-2421	ZRSR1	-0.53
bta-miR-1343-5p	FAM163A	-0.67	bta-miR-2421	ZBTB7A	-0.52
bta-miR-1343-5p	WNK2	-0.67	bta-miR-2421	CREBRF	-0.51
bta-miR-1343-5p	GPRC5A	-0.66	bta-miR-2421	GRIN2B	-0.51

bta-miR-1343-5p	CDR2L	-0.66	bta-miR-2434	WDR38	-0.94
bta-miR-1343-5p	RP11-195F19.5	-0.66	bta-miR-2434	MT-ATP8	-0.93
bta-miR-1343-5p	SNX17	-0.66	bta-miR-2434	KCNMB1	-0.8
bta-miR-1343-5p	S100A2	-0.66	bta-miR-2434	FSIP1	-0.8
bta-miR-1343-5p	CERS3	-0.66	bta-miR-2434	CYSLTR2	-0.76
bta-miR-1343-5p	PXN	-0.65	bta-miR-2434	MEI4	-0.72
bta-miR-1343-5p	CTB-96E2.2	-0.65	bta-miR-2434	UBTD2	-0.72
bta-miR-1343-5p	C19orf35	-0.65	bta-miR-2434	SLC13A3	-0.71
bta-miR-1343-5p	TAGLN	-0.65	bta-miR-2434	CRISP2	-0.7
bta-miR-1343-5p	KIF17	-0.65	bta-miR-2434	MT-ND4L	-0.68
bta-miR-1343-5p	HOXA6	-0.65	bta-miR-2434	IMPG1	-0.68
bta-miR-1343-5p	CSMD2	-0.65	bta-miR-2434	PPIL1	-0.67
bta-miR-1343-5p	UQCC1	-0.65	bta-miR-2434	NAMPT	-0.64
bta-miR-1343-5p	C17orf80	-0.64	bta-miR-2434	AC110619.2	-0.63
bta-miR-1343-5p	PAX8	-0.64	bta-miR-2434	SEC61B	-0.62
bta-miR-1343-5p	NUDC	-0.64	bta-miR-2434	TFRC	-0.62
bta-miR-1343-5p	NACC1	-0.64	bta-miR-2434	NRIP1	-0.6
bta-miR-1343-5p	AC145676.2	-0.64	bta-miR-2434	YIPF1	-0.6
bta-miR-1343-5p	CNNM4	-0.64	bta-miR-2434	FOXM1	-0.59
bta-miR-1343-5p	LMAN2L	-0.64	bta-miR-2434	RAD51B	-0.57
bta-miR-1343-5p	PLOD3	-0.64	bta-miR-2434	SYNE3	-0.57
bta-miR-1343-5p	CALCOCO1	-0.64	bta-miR-2434	ADRB2	-0.56
bta-miR-1343-5p	AC024940.1	-0.64	bta-miR-2434	C16orf92	-0.56
bta-miR-1343-5p	CD300LB	-0.64	bta-miR-2434	HRK	-0.55
bta-miR-1343-5p	EFHD2	-0.64	bta-miR-2434	DDX42	-0.55
bta-miR-1343-5p	CBX2	-0.64	bta-miR-2434	TST	-0.55
bta-miR-1343-5p	KIF21B	-0.64	bta-miR-2434	EXD1	-0.55
bta-miR-1343-5p	KIAA0247	-0.63	bta-miR-2434	HOXB8	-0.55
bta-miR-1343-5p	KDM6B	-0.63	bta-miR-2434	AL589765.1	-0.55
bta-miR-1343-5p	CYP46A1	-0.63	bta-miR-2434	ZNF770	-0.55
bta-miR-1343-5p	PPP2R4	-0.63	bta-miR-2434	TFAP2A	-0.54
bta-miR-1343-5p	FOXI2	-0.63	bta-miR-2434	DMRTC2	-0.54
bta-miR-1343-5p	CRY2	-0.62	bta-miR-2434	ZC3HAV1L	-0.54
bta-miR-1343-5p	STX6	-0.62	bta-miR-2434	FAM174A	-0.54
bta-miR-1343-5p	PITX1	-0.62	bta-miR-2434	NMUR2	-0.54
bta-miR-1343-5p	LINC00632	-0.62	bta-miR-2434	CTRC	-0.54
bta-miR-1343-5p	RNF222	-0.62	bta-miR-2434	GABARAP	-0.53
bta-miR-1343-5p	PPARGC1B	-0.62	bta-miR-2434	HOXC8	-0.53
bta-miR-1343-5p	PCYT1B	-0.62	bta-miR-2434	RP11-247C2.2	-0.53
bta-miR-1343-5p	KIRREL	-0.62	bta-miR-2434	ADGB	-0.52
bta-miR-1343-5p	EPB41	-0.62	bta-miR-2434	DNAI2	-0.52
bta-miR-1343-5p	MEA1	-0.62	bta-miR-2434	TNK1	-0.52
bta-miR-1343-5p	VSTM5	-0.61	bta-miR-2434	DPM2	-0.52
bta-miR-1343-5p	SERINC2	-0.61	bta-miR-2434	PEX11G	-0.51
bta-miR-1343-5p	NFAM1	-0.61	bta-miR-2434	KRTAP19-2	-0.5

bta-miR-1343-5p	TNS1	-0.61	bta-miR-2434	P2RY13	-0.5
bta-miR-1343-5p	PYGM	-0.61	bta-miR-2434	TP63	-0.5
bta-miR-1343-5p	TATDN3	-0.61	bta-miR-2434	SOSTDC1	-0.5
bta-miR-1343-5p	CLEC3A	-0.6	bta-miR-2444	RP4-758J18.2	-1.05
bta-miR-1343-5p	CTC-360G5.1	-0.6	bta-miR-2444	RP11-67H2.1	-0.92
bta-miR-1343-5p	SHISA9	-0.6	bta-miR-2444	GORASP1	-0.91
bta-miR-1343-5p	ERGIC1	-0.6	bta-miR-2444	RP11-105C20.2	-0.82
bta-miR-1343-5p	SLC7A5	-0.6	bta-miR-2444	OR6C65	-0.79
bta-miR-1343-5p	SNCG	-0.6	bta-miR-2444	RP11-169F17.1	-0.79
bta-miR-1343-5p	ALX4	-0.6	bta-miR-2444	AC090186.1	-0.69
bta-miR-1343-5p	AGAP2-AS1	-0.59	bta-miR-2444	GLRA3	-0.68
bta-miR-1343-5p	TNFSF12	-0.59	bta-miR-2444	TFEC	-0.66
bta-miR-1343-5p	RHOB	-0.59	bta-miR-2444	AMELY	-0.64
bta-miR-1343-5p	PPIE	-0.59	bta-miR-2444	AMELX	-0.63
bta-miR-1343-5p	KRTAP10-1	-0.59	bta-miR-2444	SMIM11	-0.62
bta-miR-1343-5p	CHST1	-0.59	bta-miR-2444	AICDA	-0.62
bta-miR-1343-5p	PVRIG	-0.59	bta-miR-2444	EI24	-0.61
bta-miR-1343-5p	KCNMB1	-0.59	bta-miR-2444	NRXN1	-0.61
bta-miR-1343-5p	MTAP	-0.59	bta-miR-2444	CMBL	-0.61
bta-miR-1343-5p	PPP2R5D	-0.59	bta-miR-2444	OR5H14	-0.6
bta-miR-1343-5p	PRRG3	-0.59	bta-miR-2444	LCMT2	-0.58
bta-miR-1343-5p	KCNC3	-0.59	bta-miR-2444	AJAP1	-0.58
bta-miR-1343-5p	UCP2	-0.58	bta-miR-2444	PLGRKT	-0.58
bta-miR-1343-5p	TAL1	-0.58	bta-miR-2444	ELAVL3	-0.57
bta-miR-1343-5p	AP001652.1	-0.58	bta-miR-2444	DRD5	-0.57
bta-miR-1343-5p	NUP98	-0.58	bta-miR-2444	ZPBP	-0.57
bta-miR-1343-5p	GATS	-0.58	bta-miR-2444	NXPE4	-0.57
bta-miR-1343-5p	AC006946.15	-0.58	bta-miR-2444	DNMT3A	-0.57
bta-miR-1343-5p	TMEM79	-0.58	bta-miR-2444	RBFOX2	-0.56
bta-miR-1343-5p	SNX12	-0.58	bta-miR-2444	AAED1	-0.56
bta-miR-1343-5p	CELSR2	-0.57	bta-miR-2444	CDC26	-0.56
bta-miR-1343-5p	KRT17	-0.57	bta-miR-2444	PTP4A2	-0.54
bta-miR-1343-5p	NHP2	-0.57	bta-miR-2444	CIR1	-0.54
bta-miR-1343-5p	DUSP28	-0.57	bta-miR-2444	AC140061.12	-0.54
bta-miR-1343-5p	RBMXL2	-0.57	bta-miR-2444	SAMD12	-0.53
bta-miR-1343-5p	PTK2B	-0.57	bta-miR-2444	TLE1	-0.53
bta-miR-1343-5p	PGS1	-0.57	bta-miR-2444	KB-1980E6.3	-0.53
bta-miR-1343-5p	CSNK2B-LY6G5B-1181	-0.57	bta-miR-2444	ELAVL4	-0.53
bta-miR-1343-5p	GABARAPL1	-0.57	bta-miR-2444	GKAP1	-0.52
bta-miR-1343-5p	SYNGR1	-0.57	bta-miR-2444	PLCXD2	-0.52
bta-miR-1343-5p	OBP2B	-0.57	bta-miR-2444	C1orf63	-0.51
bta-miR-1343-5p	LOXL1	-0.56	bta-miR-2444	AC002451.1	-0.51
bta-miR-1343-5p	HDAC10	-0.56	bta-miR-2444	C20orf85	-0.51
bta-miR-1343-5p	EMC10	-0.56	bta-miR-2444	SSMEM1	-0.51
bta-miR-1343-5p	CEACAM1	-0.56	bta-miR-2444	C2orf83	-0.51

bta-miR-1343-5p	CD7	-0.56	bta-miR-2444	ANGPTL6	-0.51
bta-miR-1343-5p	MPP2	-0.56	bta-miR-2444	RP11-1102P16.1	-0.5
bta-miR-1343-5p	ELOVL1	-0.56	bta-miR-2444	HNRNPUL1	-0.5
bta-miR-1343-5p	LZTS2	-0.56	bta-miR-2444	AC005477.1	-0.5
bta-miR-1343-5p	NYAP1	-0.56	bta-miR-2452	AC004899.1	-1.73
bta-miR-1343-5p	DCHS1	-0.56	bta-miR-2452	NTNG1	-1.29
bta-miR-1343-5p	HRH3	-0.56	bta-miR-2452	C15orf32	-1.17
bta-miR-1343-5p	CLTCL1	-0.56	bta-miR-2452	PDE7B	-1
bta-miR-1343-5p	NIPSNAP1	-0.55	bta-miR-2452	LIMK2	-1
bta-miR-1343-5p	GSC	-0.55	bta-miR-2452	ZNF23	-0.88
bta-miR-1343-5p	COL5A3	-0.55	bta-miR-2452	SMCP	-0.83
bta-miR-1343-5p	ADCYAP1R1	-0.55	bta-miR-2452	GGACT	-0.73
bta-miR-1343-5p	NOTUM	-0.55	bta-miR-2452	TMPRSS11F	-0.7
bta-miR-1343-5p	RPP30	-0.55	bta-miR-2452	SPP2	-0.66
bta-miR-1343-5p	POLR2J2	-0.55	bta-miR-2452	TBCE	-0.66
bta-miR-1343-5p	PNMA6A	-0.55	bta-miR-2452	C2orf74	-0.64
bta-miR-1343-5p	TNNT2	-0.55	bta-miR-2452	TMEM223	-0.64
bta-miR-1343-5p	ONECUT3	-0.55	bta-miR-2452	APOBEC2	-0.62
bta-miR-1343-5p	POLE3	-0.55	bta-miR-2452	SATL1	-0.62
bta-miR-1343-5p	ATXN2L	-0.55	bta-miR-2452	CRYBB1	-0.62
bta-miR-1343-5p	C10orf105	-0.55	bta-miR-2452	TMED6	-0.59
bta-miR-1343-5p	TBC1D22B	-0.55	bta-miR-2452	C17orf67	-0.59
bta-miR-1343-5p	PRSS42	-0.55	bta-miR-2452	PPAP2B	-0.56
bta-miR-1343-5p	DKFZP434O1614	-0.55	bta-miR-2452	C2orf83	-0.54
bta-miR-1343-5p	FAM101A	-0.55	bta-miR-2452	PSME4	-0.54
bta-miR-1343-5p	PPP1R32	-0.55	bta-miR-2452	CDC37	-0.54
bta-miR-1343-5p	STC1	-0.55	bta-miR-2452	ZNF785	-0.54
bta-miR-1343-5p	CYB5R3	-0.54	bta-miR-2452	C2orf88	-0.52
bta-miR-1343-5p	CCDC142	-0.54	bta-miR-2452	TAL2	-0.52
bta-miR-1343-5p	C6orf223	-0.54	bta-miR-2452	VBP1	-0.51
bta-miR-1343-5p	ATP6V0D1	-0.54	bta-miR-2452	AL138847.1	-0.5
bta-miR-1343-5p	C2orf62	-0.54	bta-miR-2452	PPP1R14B	-0.5
bta-miR-1343-5p	PPP5C	-0.54	bta-miR-2452	CHST9	-0.5
bta-miR-1343-5p	IRGQ	-0.54	bta-miR-2452	AC015987.2	-0.5
bta-miR-1343-5p	CCL22	-0.54	bta-miR-2452	TBL1Y	-0.5
bta-miR-1343-5p	ZDHHC7	-0.54	bta-miR-3613a	ZNF30	-0.88
bta-miR-1343-5p	TRIM3	-0.54	bta-miR-3613a	RNASE3	-0.88
bta-miR-1343-5p	WDTC1	-0.54	bta-miR-3613a	RNASE2	-0.88
bta-miR-1343-5p	COLQ	-0.54	bta-miR-3613a	RP11-105C20.2	-0.81
bta-miR-1343-5p	JUP	-0.54	bta-miR-3613a	AKAP6	-0.77
bta-miR-1343-5p	LEPREL4	-0.54	bta-miR-3613a	C9orf129	-0.76
bta-miR-1343-5p	NTN3	-0.54	bta-miR-3613a	EXOSC1	-0.75
bta-miR-1343-5p	DCAKD	-0.54	bta-miR-3613a	GPX4	-0.66
bta-miR-1343-5p	C1orf134	-0.53	bta-miR-3613a	ZFYVE21	-0.64
bta-miR-1343-5p	ELOVL2	-0.53	bta-miR-3613a	CTC-241N9.1	-0.59

bta-miR-1343-5p	PRKCG	-0.53	bta-miR-3613a	ANP32B	-0.59
bta-miR-1343-5p	FOXM1	-0.53	bta-miR-3613a	C2orf73	-0.57
bta-miR-1343-5p	RPH3A	-0.53	bta-miR-3613a	KRT35	-0.57
bta-miR-1343-5p	TMEM151B	-0.53	bta-miR-3613a	HIST2H4B	-0.56
bta-miR-1343-5p	R3HCC1	-0.53	bta-miR-3613a	AL162407.1	-0.54
bta-miR-1343-5p	VPS53	-0.53	bta-miR-3613a	CASP5	-0.53
bta-miR-1343-5p	CCDC149	-0.53	bta-miR-3613a	LYRM2	-0.53
bta-miR-1343-5p	LDLRAD2	-0.53	bta-miR-3613a	ZNF667	-0.52
bta-miR-1343-5p	GABBR2	-0.53	bta-miR-3613a	IFRD2	-0.52
bta-miR-1343-5p	RHBDF2	-0.53	bta-miR-3613a	AC007390.5	-0.52
bta-miR-1343-5p	CX3CL1	-0.52	bta-miR-3613a	STK38	-0.51
bta-miR-1343-5p	LBH	-0.52	bta-miR-3613a	AL590452.1	-0.51
bta-miR-1343-5p	ICAM1	-0.52	bta-miR-3613a	GATA2	-0.5
bta-miR-1343-5p	ABHD17A	-0.52	bta-miR-3613a	CYP2C9	-0.5
bta-miR-1343-5p	TBC1D16	-0.52	bta-miR-3613b	GAPVD1	-1
bta-miR-1343-5p	GDF11	-0.52	bta-miR-3613b	GAS7	-1
bta-miR-1343-5p	CNOT3	-0.52	bta-miR-3613b	USP42	-1
bta-miR-1343-5p	GINS2	-0.52	bta-miR-3613b	CCDC132	-1
bta-miR-1343-5p	ANGPTL6	-0.52	bta-miR-3613b	TMBIM6	-1
bta-miR-1343-5p	FBXO31	-0.52	bta-miR-3613b	KDELR2	-1
bta-miR-1343-5p	TUBB4A	-0.51	bta-miR-3613b	GOLT1B	-1
bta-miR-1343-5p	ZBTB46	-0.51	bta-miR-3613b	SIK2	-1
bta-miR-1343-5p	SH3TC2	-0.51	bta-miR-3613b	UBR2	-1
bta-miR-1343-5p	ATP5D	-0.51	bta-miR-3613b	KPNA6	-1
bta-miR-1343-5p	RAX	-0.51	bta-miR-3613b	MFN2	-1
bta-miR-1343-5p	RNF165	-0.51	bta-miR-3613b	HSD17B12	-1
bta-miR-1343-5p	MMP24	-0.51	bta-miR-3613b	DEDD	-1
bta-miR-1343-5p	SELRC1	-0.51	bta-miR-3613b	CDK12	-1
bta-miR-1343-5p	LPCAT3	-0.51	bta-miR-3613b	IKZF4	-1
bta-miR-1343-5p	VAT1	-0.51	bta-miR-3613b	LONRF2	-1
bta-miR-1343-5p	USP46	-0.51	bta-miR-3613b	RHOQ	-1
bta-miR-1343-5p	SZT2	-0.51	bta-miR-3613b	LGI2	-1
bta-miR-1343-5p	CTRL	-0.51	bta-miR-3613b	FAM126A	-1
bta-miR-1343-5p	SLC16A2	-0.51	bta-miR-3613b	ZFP37	-1
bta-miR-1343-5p	C11orf42	-0.51	bta-miR-3613b	SNX30	-1
bta-miR-1343-5p	TOM1L2	-0.5	bta-miR-3613b	GLE1	-1
bta-miR-1343-5p	PLEKHG4	-0.5	bta-miR-3613b	ZIC5	-1
bta-miR-1343-5p	KRTAP10-9	-0.5	bta-miR-3613b	AGAP2	-1
bta-miR-1343-5p	THY1	-0.5	bta-miR-3613b	RBM25	-1
bta-miR-1343-5p	PRR11	-0.5	bta-miR-3613b	ARHGAP35	-1
bta-miR-1343-5p	GRINA	-0.5	bta-miR-3613b	MRRF	-1
bta-miR-1343-5p	GIPR	-0.5	bta-miR-3613b	USP38	-1
bta-miR-1343-5p	TEX35	-0.5	bta-miR-3613b	CPD	-1
bta-miR-1343-5p	GAS2L2	-0.5	bta-miR-3613b	CEP128	-1
bta-miR-1343-5p	TRPM4	-0.5	bta-miR-3613b	CELF2	-0.92

bta-miR-1343-5p	TMEM222	-0.5	bta-miR-3613b	FGFBP2	-0.54
bta-miR-1343-5p	C9orf171	-0.5	bta-miR-455-3p	SLC25A3	-0.71
bta-miR-1343-5p	BAHD1	-0.5	bta-miR-455-3p	PSMA2	-0.63
bta-miR-1343-5p	TPRG1L	-0.5	bta-miR-455-3p	HOXC4	-0.57
bta-miR-1343-5p	UROC1	-0.5	bta-miR-455-3p	NDUFA2	-0.56
bta-miR-1343-5p	PROSC	-0.5	bta-miR-455-3p	CUL3	-0.54
bta-miR-1343-5p	RCN3	-0.5	bta-miR-455-3p	INIP	-0.53
bta-miR-1343-5p	DPF1	-0.5	bta-miR-455-3p	TSPAN18	-0.51
bta-miR-1343-5p	SH2B1	-0.5	bta-miR-455-3p	COLEC12	-0.5
bta-miR-1343-5p	RASD2	-0.5	bta-miR-455-3p	SSR1	-0.5
bta-miR-1343-5p	RALB	-0.5	bta-miR-628	LL22NC03-63E9.3	-1.19
bta-miR-2325a	LRRC4C	-0.88	bta-miR-628	POC1B-GALNT4	-1.16
bta-miR-2325a	SYNE2	-0.56	bta-miR-628	PGBD4	-1.11
bta-miR-2325a	PTMA	-0.56	bta-miR-628	BOD1L2	-0.95
bta-miR-2325a	SCAF4	-0.52	bta-miR-628	GALNT4	-0.82
bta-miR-2325a	FAM155A	-0.5	bta-miR-628	KATNAL2	-0.79
bta-miR-2325c	TTN	-1.52	bta-miR-628	ADH7	-0.78
bta-miR-2325c	SMAD4	-1	bta-miR-628	COL9A3	-0.72
bta-miR-2325c	C7orf66	-0.89	bta-miR-628	CHST9	-0.72
bta-miR-2325c	RSPO3	-0.88	bta-miR-628	TAS2R14	-0.72
bta-miR-2325c	NECAB1	-0.87	bta-miR-628	NBPF16	-0.7
bta-miR-2325c	BOD1L2	-0.87	bta-miR-628	GALNT9	-0.69
bta-miR-2325c	RP11-595B24.2	-0.85	bta-miR-628	SPATA33	-0.69
bta-miR-2325c	ELAVL4	-0.84	bta-miR-628	STEAP1B	-0.68
bta-miR-2325c	CACNG2	-0.73	bta-miR-628	AC013269.5	-0.67
bta-miR-2325c	U2AF1	-0.69	bta-miR-628	AP3B2	-0.66
bta-miR-2325c	PTMA	-0.68	bta-miR-628	ZNF277	-0.65
bta-miR-2325c	NRXN3	-0.66	bta-miR-628	FABP1	-0.64
bta-miR-2325c	AGBL3	-0.64	bta-miR-628	SNRPA1	-0.63
bta-miR-2325c	MTMR8	-0.59	bta-miR-628	EAF1	-0.62
bta-miR-2325c	MUC19	-0.58	bta-miR-628	C8orf22	-0.61
bta-miR-2325c	ZNF30	-0.58	bta-miR-628	DALRD3	-0.6
bta-miR-2325c	CTD-2054N24.2	-0.57	bta-miR-628	SDIM1	-0.6
bta-miR-2325c	FAM27E1	-0.57	bta-miR-628	CST3	-0.59
bta-miR-2325c	FAM27E3	-0.57	bta-miR-628	HRNR	-0.58
bta-miR-2325c	OR5H14	-0.56	bta-miR-628	GOLT1B	-0.58
bta-miR-2325c	FAM27E2	-0.55	bta-miR-628	CCDC146	-0.58
bta-miR-2325c	SMIM11	-0.55	bta-miR-628	ZBTB37	-0.57
bta-miR-2325c	ZG16	-0.54	bta-miR-628	ATG4A	-0.57
bta-miR-2325c	EMC4	-0.53	bta-miR-628	PDCD5	-0.57
bta-miR-2325c	FAM185A	-0.51	bta-miR-628	ZGLP1	-0.56
bta-miR-2325c	CTB-186H2.3	-0.51	bta-miR-628	SCRN3	-0.56
bta-miR-2325c	PLN	-0.51	bta-miR-628	CLMP	-0.56
bta-miR-2325c	RPN1	-0.51	bta-miR-628	TMEM57	-0.54
bta-miR-2325c	DKFZP779J2370	-0.5	bta-miR-628	SERPINA5	-0.54

bta-miR-2359	RP4-758J18.2	-1.17	bta-miR-628	DMC1	-0.54
bta-miR-2359	SOX6	-1	bta-miR-628	FAN1	-0.53
bta-miR-2359	CDC123	-0.72	bta-miR-628	EXOSC10	-0.53
bta-miR-2359	SPINK13	-0.71	bta-miR-628	NADK	-0.53
bta-miR-2359	S100A7	-0.67	bta-miR-628	DCX	-0.53
bta-miR-2359	CCT6B	-0.64	bta-miR-628	TRAF5	-0.53
bta-miR-2359	S100A7L2	-0.62	bta-miR-628	TRIM9	-0.52
bta-miR-2359	COX6A2	-0.61	bta-miR-628	TMEM200C	-0.52
bta-miR-2359	MRPS18B	-0.58	bta-miR-628	DAOA	-0.52
bta-miR-2359	ELANE	-0.56	bta-miR-628	CDC25C	-0.52
bta-miR-2359	OSTN	-0.56	bta-miR-628	RNF34	-0.51
bta-miR-2359	RP11-67H2.1	-0.54	bta-miR-628	ZFP41	-0.51
bta-miR-2359	TMEFF2	-0.53	bta-miR-628	CST9L	-0.51
bta-miR-2359	LAMTOR3	-0.52	bta-miR-628	FUNDC2	-0.51
bta-miR-2359	SERPINB5	-0.51	bta-miR-628	AC074091.13	-0.51
bta-miR-2359	DCAF4L2	-0.5	bta-miR-628	DOK2	-0.51
bta-miR-2393	NBPF16	-1.05	bta-miR-628	MORC4	-0.51
bta-miR-2393	C12orf36	-1.04	bta-miR-628	NNT	-0.5
			bta-miR-628	KRT31	-0.5

Table S2. Predicted mRNA targets of miRNAs differentially expressed between 8-cell SG SM vs. 8-Cell FG SM.

miRNAs	Genes	Cumulative Context Score	miRNAs	Genes	Cumulative Context Score
bta-miR-1281	HIST2H3A	-1.22	bta-miR-1343-5p	TUBB4A	-0.51
bta-miR-1281	AL592284.1	-1	bta-miR-1343-5p	ZBTB46	-0.51
bta-miR-1281	C22orf46	-0.96	bta-miR-1343-5p	SH3TC2	-0.51
bta-miR-1281	SLC11A1	-0.94	bta-miR-1343-5p	ATP5D	-0.51
bta-miR-1281	GAB2	-0.89	bta-miR-1343-5p	RAX	-0.51
bta-miR-1281	MANBAL	-0.83	bta-miR-1343-5p	RNF165	-0.51
bta-miR-1281	LTB	-0.83	bta-miR-1343-5p	MMP24	-0.51
bta-miR-1281	MSANTD1	-0.8	bta-miR-1343-5p	SELRC1	-0.51
bta-miR-1281	LIF	-0.8	bta-miR-1343-5p	LPCAT3	-0.51
bta-miR-1281	TEX22	-0.8	bta-miR-1343-5p	VAT1	-0.51
bta-miR-1281	FAM98A	-0.78	bta-miR-1343-5p	USP46	-0.51
bta-miR-1281	CTD-3214H19.16	-0.78	bta-miR-1343-5p	SZT2	-0.51
bta-miR-1281	ADAMTS13	-0.78	bta-miR-1343-5p	CTRL	-0.51
bta-miR-1281	AGFG2	-0.76	bta-miR-1343-5p	SLC16A2	-0.51
bta-miR-1281	DDX49	-0.76	bta-miR-1343-5p	C11orf42	-0.51
bta-miR-1281	USP39	-0.69	bta-miR-1343-5p	TOM1L2	-0.5
bta-miR-1281	ZCCHC24	-0.69	bta-miR-1343-5p	PLEKHG4	-0.5
bta-miR-1281	SLC25A21	-0.65	bta-miR-1343-5p	KRTAP10-9	-0.5
bta-miR-1281	CELF1	-0.64	bta-miR-1343-5p	THY1	-0.5
bta-miR-1281	SLC35B2	-0.64	bta-miR-1343-5p	PRR11	-0.5

bta-miR-1281	FAM170B	-0.64	bta-miR-1343-5p	GRINA	-0.5
bta-miR-1281	PRX	-0.64	bta-miR-1343-5p	GIPR	-0.5
bta-miR-1281	GPR26	-0.63	bta-miR-1343-5p	TEX35	-0.5
bta-miR-1281	APOL6	-0.63	bta-miR-1343-5p	GAS2L2	-0.5
bta-miR-1281	LSM12	-0.6	bta-miR-1343-5p	TRPM4	-0.5
bta-miR-1281	KLHL30	-0.59	bta-miR-1343-5p	TMEM222	-0.5
bta-miR-1281	BMP8B	-0.59	bta-miR-1343-5p	C9orf171	-0.5
bta-miR-1281	AC074212.3	-0.59	bta-miR-1343-5p	BAHD1	-0.5
bta-miR-1281	IDH2	-0.58	bta-miR-1343-5p	TPRG1L	-0.5
bta-miR-1281	SH3GLB2	-0.58	bta-miR-1343-5p	UROC1	-0.5
bta-miR-1281	FLJ27365	-0.58	bta-miR-1343-5p	PROSC	-0.5
bta-miR-1281	PAQR6	-0.57	bta-miR-1343-5p	RCN3	-0.5
bta-miR-1281	TSPO2	-0.57	bta-miR-1343-5p	DPF1	-0.5
bta-miR-1281	FLJ00418	-0.56	bta-miR-1343-5p	SH2B1	-0.5
bta-miR-1281	PHB2	-0.56	bta-miR-1343-5p	RASD2	-0.5
bta-miR-1281	SCAMP3	-0.56	bta-miR-1343-5p	RALB	-0.5
bta-miR-1281	GRK5	-0.56	bta-miR-2885	RGMA	-1.23
bta-miR-1281	RIMS4	-0.55	bta-miR-2885	WNK2	-1.13
bta-miR-1281	ZNF70	-0.55	bta-miR-2885	PLA2G1B	-1.08
bta-miR-1281	BOK	-0.55	bta-miR-2885	SLC25A28	-1.02
bta-miR-1281	NSG2	-0.54	bta-miR-2885	YIF1B	-0.95
bta-miR-1281	KAZALD1	-0.54	bta-miR-2885	AGFG2	-0.95
bta-miR-1281	CNIH3	-0.54	bta-miR-2885	RAX	-0.91
bta-miR-1281	FBXW7	-0.54	bta-miR-2885	MZF1	-0.86
bta-miR-1281	AC005609.1	-0.53	bta-miR-2885	FAM73B	-0.86
bta-miR-1281	NAB2	-0.53	bta-miR-2885	LENG8	-0.85
bta-miR-1281	SLC39A11	-0.53	bta-miR-2885	LTBP4	-0.83
bta-miR-1281	BHLHE22	-0.52	bta-miR-2885	CERS1	-0.83
bta-miR-1281	KLHL29	-0.52	bta-miR-2885	CABP1	-0.83
bta-miR-1281	PMFBP1	-0.52	bta-miR-2885	MARK4	-0.83
bta-miR-1281	E4F1	-0.52	bta-miR-2885	AL450307.1	-0.82
bta-miR-1281	CLPP	-0.52	bta-miR-2885	VAMP8	-0.81
bta-miR-1281	NFATC4	-0.52	bta-miR-2885	SPSB4	-0.81
bta-miR-1281	LDB2	-0.51	bta-miR-2885	NKX2-5	-0.8
bta-miR-1281	C1orf64	-0.51	bta-miR-2885	CCDC64	-0.8
bta-miR-1281	ZBTB7C	-0.51	bta-miR-2885	C3orf20	-0.79
bta-miR-1281	ZNF575	-0.5	bta-miR-2885	DKFZP779J2370	-0.78
bta-miR-1281	NKX6-3	-0.5	bta-miR-2885	UBALD1	-0.76
bta-miR-1281	AHRR	-0.5	bta-miR-2885	FAM219A	-0.75
bta-miR-1343-5p	PRX	-1.51	bta-miR-2885	PTBP1	-0.75
bta-miR-1343-5p	KIAA0513	-1.48	bta-miR-2885	PACSIN2	-0.75
bta-miR-1343-5p	KSR2	-1.29	bta-miR-2885	AL590822.1	-0.75

bta-miR-1343-5p	RAB37	-1.27	bta-miR-2885	PEG10	-0.74
bta-miR-1343-5p	LY6G6C	-1.24	bta-miR-2885	FTCDNL1	-0.73
bta-miR-1343-5p	EHD2	-1.22	bta-miR-2885	MIB2	-0.73
bta-miR-1343-5p	C17orf103	-1.13	bta-miR-2885	ATAD3B	-0.73
bta-miR-1343-5p	GAS8	-1.1	bta-miR-2885	IQSEC2	-0.72
bta-miR-1343-5p	RGMA	-1.03	bta-miR-2885	MAP2K3	-0.71
bta-miR-1343-5p	OSM	-1	bta-miR-2885	HDAC11	-0.71
bta-miR-1343-5p	LYPD1	-0.95	bta-miR-2885	C17orf103	-0.71
bta-miR-1343-5p	PKD1	-0.95	bta-miR-2885	ACVR1B	-0.71
bta-miR-1343-5p	MS4A15	-0.95	bta-miR-2885	PTCD2	-0.71
bta-miR-1343-5p	GPA33	-0.95	bta-miR-2885	AC002472.13	-0.7
bta-miR-1343-5p	COTL1	-0.92	bta-miR-2885	FLJ45079	-0.7
bta-miR-1343-5p	FKBP1A	-0.91	bta-miR-2885	REPIN1	-0.7
bta-miR-1343-5p	HOXB5	-0.91	bta-miR-2885	REXO2	-0.69
bta-miR-1343-5p	SPSB1	-0.86	bta-miR-2885	FOSB	-0.68
bta-miR-1343-5p	TFCP2L1	-0.86	bta-miR-2885	GTF2IRD1	-0.68
bta-miR-1343-5p	TFEB	-0.86	bta-miR-2885	TFAP2A	-0.68
bta-miR-1343-5p	FHL3	-0.86	bta-miR-2885	TXNRD3	-0.67
bta-miR-1343-5p	SIPA1L3	-0.85	bta-miR-2885	CTC1	-0.67
bta-miR-1343-5p	LDLRAP1	-0.84	bta-miR-2885	ZNF827	-0.67
bta-miR-1343-5p	KCTD17	-0.83	bta-miR-2885	CTXN1	-0.67
bta-miR-1343-5p	SIX5	-0.83	bta-miR-2885	PI4KB	-0.67
bta-miR-1343-5p	KB-1507C5.2	-0.82	bta-miR-2885	PRKAR1B	-0.66
bta-miR-1343-5p	MVB12B	-0.82	bta-miR-2885	KLHL18	-0.66
bta-miR-1343-5p	ATG9A	-0.8	bta-miR-2885	EXTL1	-0.66
bta-miR-1343-5p	HMGA1	-0.8	bta-miR-2885	MIDN	-0.66
bta-miR-1343-5p	TBC1D13	-0.79	bta-miR-2885	MEX3B	-0.65
bta-miR-1343-5p	GRB7	-0.79	bta-miR-2885	TOX2	-0.65
bta-miR-1343-5p	KLK14	-0.78	bta-miR-2885	EDNRA	-0.65
bta-miR-1343-5p	UBE2QL1	-0.78	bta-miR-2885	C20orf26	-0.65
bta-miR-1343-5p	THTPA	-0.77	bta-miR-2885	DOLPP1	-0.64
bta-miR-1343-5p	PIRT	-0.77	bta-miR-2885	PCGF3	-0.64
bta-miR-1343-5p	TNS4	-0.77	bta-miR-2885	VPS37B	-0.64
bta-miR-1343-5p	HEPACAM	-0.77	bta-miR-2885	HSPB6	-0.63
bta-miR-1343-5p	STIM1	-0.77	bta-miR-2885	TWIST2	-0.62
bta-miR-1343-5p	RRP1	-0.76	bta-miR-2885	SHISA6	-0.62
bta-miR-1343-5p	PACRG	-0.76	bta-miR-2885	ICOSLG	-0.62
bta-miR-1343-5p	PSORS1C1	-0.76	bta-miR-2885	ATP13A2	-0.62
bta-miR-1343-5p	GAREML	-0.76	bta-miR-2885	AC026703.1	-0.61
bta-miR-1343-5p	NUPR1	-0.75	bta-miR-2885	TLR4	-0.61
bta-miR-1343-5p	SERPINE3	-0.75	bta-miR-2885	LEMD2	-0.61
bta-miR-1343-5p	AQP5	-0.75	bta-miR-2885	VAT1L	-0.6

bta-miR-1343-5p	TSTA3	-0.75	bta-miR-2885	CDK3	-0.6
bta-miR-1343-5p	GLIS1	-0.75	bta-miR-2885	FOXK1	-0.6
bta-miR-1343-5p	SEC14L6	-0.74	bta-miR-2885	CRTC1	-0.6
bta-miR-1343-5p	LY6E	-0.74	bta-miR-2885	TUFT1	-0.6
bta-miR-1343-5p	CAMKV	-0.74	bta-miR-2885	RP4-559A3.7	-0.59
bta-miR-1343-5p	TMEM150A	-0.73	bta-miR-2885	CHMP7	-0.59
bta-miR-1343-5p	TMCC2	-0.73	bta-miR-2885	HRAS	-0.59
bta-miR-1343-5p	SCAND1	-0.73	bta-miR-2885	TMEM129	-0.59
bta-miR-1343-5p	LOH12CR1	-0.72	bta-miR-2885	EEPD1	-0.58
bta-miR-1343-5p	IL17REL	-0.72	bta-miR-2885	MYO9B	-0.58
bta-miR-1343-5p	TMEM27	-0.71	bta-miR-2885	ZFP36L1	-0.58
bta-miR-1343-5p	PRRT2	-0.71	bta-miR-2885	MVB12B	-0.58
bta-miR-1343-5p	ABHD14B	-0.71	bta-miR-2885	MAP2K7	-0.57
bta-miR-1343-5p	RNASEH2A	-0.71	bta-miR-2885	GORASP1	-0.57
bta-miR-1343-5p	JPH4	-0.71	bta-miR-2885	TTYH3	-0.57
bta-miR-1343-5p	NDRG4	-0.71	bta-miR-2885	TRPC5	-0.57
bta-miR-1343-5p	RARG	-0.7	bta-miR-2885	RASA4	-0.56
bta-miR-1343-5p	LIF	-0.7	bta-miR-2885	OAF	-0.56
bta-miR-1343-5p	CABP7	-0.7	bta-miR-2885	APOC4	-0.56
bta-miR-1343-5p	RIMS4	-0.7	bta-miR-2885	NCS1	-0.56
bta-miR-1343-5p	AIF1L	-0.7	bta-miR-2885	C10orf105	-0.56
bta-miR-1343-5p	PTMS	-0.7	bta-miR-2885	LRRC8A	-0.56
bta-miR-1343-5p	CASQ1	-0.7	bta-miR-2885	ARHGAP35	-0.56
bta-miR-1343-5p	SERPINB6	-0.7	bta-miR-2885	PDCD5	-0.56
bta-miR-1343-5p	GJB4	-0.7	bta-miR-2885	CBX2	-0.56
bta-miR-1343-5p	KIAA1644	-0.7	bta-miR-2885	RPS6KA2	-0.56
bta-miR-1343-5p	FAM178B	-0.69	bta-miR-2885	CORO7	-0.55
bta-miR-1343-5p	GPIHBP1	-0.69	bta-miR-2885	ATG4B	-0.55
bta-miR-1343-5p	BLOC1S3	-0.68	bta-miR-2885	CRB2	-0.55
bta-miR-1343-5p	FAM222B	-0.68	bta-miR-2885	MOCS1	-0.55
bta-miR-1343-5p	SLC6A8	-0.68	bta-miR-2885	CCHCR1	-0.55
bta-miR-1343-5p	USP21	-0.68	bta-miR-2885	JPH4	-0.54
bta-miR-1343-5p	ZNF703	-0.68	bta-miR-2885	BRSK2	-0.54
bta-miR-1343-5p	VAMP2	-0.67	bta-miR-2885	SBK2	-0.54
bta-miR-1343-5p	ASTN1	-0.67	bta-miR-2885	FAM126A	-0.53
bta-miR-1343-5p	FCRL6	-0.67	bta-miR-2885	RBM38	-0.53
bta-miR-1343-5p	APLNR	-0.67	bta-miR-2885	CAMK2N2	-0.53
bta-miR-1343-5p	DDX41	-0.67	bta-miR-2885	ATG16L1	-0.53
bta-miR-1343-5p	FAM163A	-0.67	bta-miR-2885	MEGF8	-0.53
bta-miR-1343-5p	WNK2	-0.67	bta-miR-2885	CYTH1	-0.52
bta-miR-1343-5p	GPRC5A	-0.66	bta-miR-2885	TMEM222	-0.52
bta-miR-1343-5p	CDR2L	-0.66	bta-miR-2885	MEF2A	-0.52

bta-miR-1343-5p	RP11-195F19.5	-0.66	bta-miR-2885	ASIC3	-0.52
bta-miR-1343-5p	SNX17	-0.66	bta-miR-2885	C19orf26	-0.52
bta-miR-1343-5p	S100A2	-0.66	bta-miR-2885	FAM155A	-0.52
bta-miR-1343-5p	CERS3	-0.66	bta-miR-2885	SEMA4D	-0.51
bta-miR-1343-5p	PXN	-0.65	bta-miR-2885	HIC2	-0.51
bta-miR-1343-5p	CTB-96E2.2	-0.65	bta-miR-2885	CST5	-0.51
bta-miR-1343-5p	C19orf35	-0.65	bta-miR-2885	KLHL26	-0.51
bta-miR-1343-5p	TAGLN	-0.65	bta-miR-2885	ATP6V0C	-0.51
bta-miR-1343-5p	KIF17	-0.65	bta-miR-2885	MSRB3	-0.51
bta-miR-1343-5p	HOXA6	-0.65	bta-miR-2885	MUSTN1	-0.51
bta-miR-1343-5p	CSMD2	-0.65	bta-miR-2885	SMG6	-0.51
bta-miR-1343-5p	UQCC1	-0.65	bta-miR-2885	CALY	-0.51
bta-miR-1343-5p	C17orf80	-0.64	bta-miR-2885	TCN2	-0.51
bta-miR-1343-5p	PAX8	-0.64	bta-miR-2885	KIF21B	-0.51
bta-miR-1343-5p	NUDC	-0.64	bta-miR-2885	EZR	-0.5
bta-miR-1343-5p	NACC1	-0.64	bta-miR-2885	CACNA2D2	-0.5
bta-miR-1343-5p	AC145676.2	-0.64	bta-miR-2885	KLF13	-0.5
bta-miR-1343-5p	CNNM4	-0.64	bta-miR-2885	ONECUT3	-0.5
bta-miR-1343-5p	LMAN2L	-0.64	bta-miR-2885	NRARP	-0.5
bta-miR-1343-5p	PLOD3	-0.64	bta-miR-2885	PRR18	-0.5
bta-miR-1343-5p	CALCOCO1	-0.64	bta-miR-2885	ADRA2B	-0.5
bta-miR-1343-5p	AC024940.1	-0.64	bta-miR-2885	KIAA0556	-0.5
bta-miR-1343-5p	CD300LB	-0.64	bta-miR-2885	PAX2	-0.5
bta-miR-1343-5p	EFHD2	-0.64	bta-miR-450b	CAMK2N1	-1.01
bta-miR-1343-5p	CBX2	-0.64	bta-miR-450b	C2orf74	-0.67
bta-miR-1343-5p	KIF21B	-0.64	bta-miR-450b	RP11-169F17.1	-0.67
bta-miR-1343-5p	KIAA0247	-0.63	bta-miR-450b	CHMP2B	-0.66
bta-miR-1343-5p	KDM6B	-0.63	bta-miR-450b	RAB40A	-0.64
bta-miR-1343-5p	CYP46A1	-0.63	bta-miR-450b	XRCC4	-0.63
bta-miR-1343-5p	PPP2R4	-0.63	bta-miR-450b	CT62	-0.62
bta-miR-1343-5p	FOXI2	-0.63	bta-miR-450b	RAET1L	-0.62
bta-miR-1343-5p	CRY2	-0.62	bta-miR-450b	SPTSSB	-0.6
bta-miR-1343-5p	STX6	-0.62	bta-miR-450b	UBD	-0.59
bta-miR-1343-5p	PITX1	-0.62	bta-miR-450b	ZNF23	-0.57
bta-miR-1343-5p	LINC00632	-0.62	bta-miR-450b	CDKN2C	-0.57
bta-miR-1343-5p	RNF222	-0.62	bta-miR-450b	PARK7	-0.55
bta-miR-1343-5p	PPARGC1B	-0.62	bta-miR-450b	SLC17A6	-0.55
bta-miR-1343-5p	PCYT1B	-0.62	bta-miR-450b	C15orf32	-0.54
bta-miR-1343-5p	KIRREL	-0.62	bta-miR-450b	B2M	-0.53
bta-miR-1343-5p	EPB41	-0.62	bta-miR-450b	TFRC	-0.53
bta-miR-1343-5p	MEA1	-0.62	bta-miR-450b	TRO	-0.51
bta-miR-1343-5p	VSTM5	-0.61	bta-miR-760-5p	CD300LB	-1.29

bta-miR-1343-5p	SERINC2	-0.61	bta-miR-760-5p	GORASP1	-1.27
bta-miR-1343-5p	NFAM1	-0.61	bta-miR-760-5p	AC016722.1	-0.99
bta-miR-1343-5p	TNS1	-0.61	bta-miR-760-5p	CT62	-0.98
bta-miR-1343-5p	PYGM	-0.61	bta-miR-760-5p	GLIPR2	-0.93
bta-miR-1343-5p	TATDN3	-0.61	bta-miR-760-5p	SLC4A11	-0.9
bta-miR-1343-5p	CLEC3A	-0.6	bta-miR-760-5p	RHOU	-0.87
bta-miR-1343-5p	CTC-360G5.1	-0.6	bta-miR-760-5p	OR5AU1	-0.85
bta-miR-1343-5p	SHISA9	-0.6	bta-miR-760-5p	PNKD	-0.84
bta-miR-1343-5p	ERGIC1	-0.6	bta-miR-760-5p	OR1L3	-0.84
bta-miR-1343-5p	SLC7A5	-0.6	bta-miR-760-5p	DOCK1	-0.82
bta-miR-1343-5p	SNCG	-0.6	bta-miR-760-5p	PRPH2	-0.81
bta-miR-1343-5p	ALX4	-0.6	bta-miR-760-5p	DMKN	-0.78
bta-miR-1343-5p	AGAP2-AS1	-0.59	bta-miR-760-5p	DNAJC15	-0.77
bta-miR-1343-5p	TNFSF12	-0.59	bta-miR-760-5p	LRP11	-0.76
bta-miR-1343-5p	RHOB	-0.59	bta-miR-760-5p	POSTN	-0.75
bta-miR-1343-5p	PPIE	-0.59	bta-miR-760-5p	CYHR1	-0.75
bta-miR-1343-5p	KRTAP10-1	-0.59	bta-miR-760-5p	SLC25A45	-0.74
bta-miR-1343-5p	CHST1	-0.59	bta-miR-760-5p	AC004899.1	-0.73
bta-miR-1343-5p	PVRIG	-0.59	bta-miR-760-5p	TMED4	-0.73
bta-miR-1343-5p	KCNMB1	-0.59	bta-miR-760-5p	POLR2J2	-0.73
bta-miR-1343-5p	MTAP	-0.59	bta-miR-760-5p	KCTD11	-0.72
bta-miR-1343-5p	PPP2R5D	-0.59	bta-miR-760-5p	PAX8	-0.72
bta-miR-1343-5p	PRRG3	-0.59	bta-miR-760-5p	SLC26A10	-0.71
bta-miR-1343-5p	KCNC3	-0.59	bta-miR-760-5p	ZFP36	-0.7
bta-miR-1343-5p	UCP2	-0.58	bta-miR-760-5p	LTA	-0.68
bta-miR-1343-5p	TAL1	-0.58	bta-miR-760-5p	DDIT4	-0.67
bta-miR-1343-5p	AP001652.1	-0.58	bta-miR-760-5p	ELAVL4	-0.67
bta-miR-1343-5p	NUP98	-0.58	bta-miR-760-5p	ZNF3	-0.67
bta-miR-1343-5p	GATS	-0.58	bta-miR-760-5p	APH1A	-0.67
bta-miR-1343-5p	AC006946.15	-0.58	bta-miR-760-5p	RPGRIP1	-0.67
bta-miR-1343-5p	TMEM79	-0.58	bta-miR-760-5p	C14orf132	-0.66
bta-miR-1343-5p	SNX12	-0.58	bta-miR-760-5p	FAM109A	-0.66
bta-miR-1343-5p	CELSR2	-0.57	bta-miR-760-5p	KRTAP9-7	-0.65
bta-miR-1343-5p	KRT17	-0.57	bta-miR-760-5p	SEC13	-0.64
bta-miR-1343-5p	NHP2	-0.57	bta-miR-760-5p	PON2	-0.64
bta-miR-1343-5p	DUSP28	-0.57	bta-miR-760-5p	GEMIN2	-0.63
bta-miR-1343-5p	RBMXL2	-0.57	bta-miR-760-5p	TBC1D10C	-0.63
bta-miR-1343-5p	PTK2B	-0.57	bta-miR-760-5p	SLC25A48	-0.62
bta-miR-1343-5p	PGS1	-0.57	bta-miR-760-5p	WFDC2	-0.61
bta-miR-1343-5p	CSNK2B-LY6G5B-1181	-0.57	bta-miR-760-5p	ESM1	-0.59
bta-miR-1343-5p	GABARAPL1	-0.57	bta-miR-760-5p	ILF2	-0.58
bta-miR-1343-5p	SYNGR1	-0.57	bta-miR-760-5p	NAT9	-0.58

bta-miR-1343-5p	OBP2B	-0.57	bta-miR-760-5p	ICAM2	-0.58
bta-miR-1343-5p	LOXL1	-0.56	bta-miR-760-5p	SLC29A2	-0.58
bta-miR-1343-5p	HDAC10	-0.56	bta-miR-760-5p	SYP	-0.57
bta-miR-1343-5p	EMC10	-0.56	bta-miR-760-5p	ACR	-0.57
bta-miR-1343-5p	CEACAM1	-0.56	bta-miR-760-5p	GDE1	-0.57
bta-miR-1343-5p	CD7	-0.56	bta-miR-760-5p	HPDL	-0.57
bta-miR-1343-5p	MPP2	-0.56	bta-miR-760-5p	RP11-144F15.1	-0.56
bta-miR-1343-5p	ELOVL1	-0.56	bta-miR-760-5p	ARFRP1	-0.56
bta-miR-1343-5p	LZTS2	-0.56	bta-miR-760-5p	C16orf91	-0.56
bta-miR-1343-5p	NYAP1	-0.56	bta-miR-760-5p	CHP2	-0.56
bta-miR-1343-5p	DCHS1	-0.56	bta-miR-760-5p	BCL2L2	-0.56
bta-miR-1343-5p	HRH3	-0.56	bta-miR-760-5p	C12orf5	-0.56
bta-miR-1343-5p	CLTCL1	-0.56	bta-miR-760-5p	GOT1	-0.55
bta-miR-1343-5p	NIPSNAP1	-0.55	bta-miR-760-5p	PCBD2	-0.55
bta-miR-1343-5p	GSC	-0.55	bta-miR-760-5p	DLK2	-0.55
bta-miR-1343-5p	COL5A3	-0.55	bta-miR-760-5p	CDK6	-0.54
bta-miR-1343-5p	ADCYAP1R1	-0.55	bta-miR-760-5p	CACNG8	-0.54
bta-miR-1343-5p	NOTUM	-0.55	bta-miR-760-5p	TMEM169	-0.54
bta-miR-1343-5p	RPP30	-0.55	bta-miR-760-5p	AL450307.1	-0.54
bta-miR-1343-5p	POLR2J2	-0.55	bta-miR-760-5p	RAD54B	-0.54
bta-miR-1343-5p	PNMA6A	-0.55	bta-miR-760-5p	OR10A4	-0.53
bta-miR-1343-5p	TNNT2	-0.55	bta-miR-760-5p	ENDOV	-0.53
bta-miR-1343-5p	ONECUT3	-0.55	bta-miR-760-5p	MRPS18B	-0.53
bta-miR-1343-5p	POLE3	-0.55	bta-miR-760-5p	GIMAP6	-0.53
bta-miR-1343-5p	ATXN2L	-0.55	bta-miR-760-5p	TWIST1	-0.53
bta-miR-1343-5p	C10orf105	-0.55	bta-miR-760-5p	C17orf64	-0.52
bta-miR-1343-5p	TBC1D22B	-0.55	bta-miR-760-5p	DAPP1	-0.52
bta-miR-1343-5p	PRSS42	-0.55	bta-miR-760-5p	JPH2	-0.52
bta-miR-1343-5p	DKFZP434O1614	-0.55	bta-miR-760-5p	STMN4	-0.52
bta-miR-1343-5p	FAM101A	-0.55	bta-miR-760-5p	SH2D2A	-0.51
bta-miR-1343-5p	PPP1R32	-0.55	bta-miR-760-5p	MLANA	-0.51
bta-miR-1343-5p	STC1	-0.55	bta-miR-760-5p	ARHGEF2	-0.5
bta-miR-1343-5p	CYB5R3	-0.54	bta-miR-760-5p	WNT9B	-0.5
bta-miR-1343-5p	CCDC142	-0.54	bta-miR-760-5p	KDM1A	-0.5
bta-miR-1343-5p	C6orf223	-0.54	bta-miR-760-5p	ASIC4	-0.5
bta-miR-1343-5p	ATP6V0D1	-0.54	bta-miR-760-5p	PRNT	-0.5
bta-miR-1343-5p	C2orf62	-0.54	bta-miR-760-5p	RASSF2	-0.5
bta-miR-1343-5p	PPP5C	-0.54	bta-miR-760-5p	OR4M2	-0.5
bta-miR-1343-5p	IRGQ	-0.54	bta-miR-760-5p	BCL2L1	-0.5
bta-miR-1343-5p	CCL22	-0.54	bta-miR-3613b	HSD17B12	-1
bta-miR-1343-5p	ZDHHC7	-0.54	bta-miR-3613b	LONRF2	-1
bta-miR-1343-5p	TRIM3	-0.54	bta-miR-3613b	MFN2	-1

bta-miR-1343-5p	WDTC1	-0.54	bta-miR-3613b	RHOQ	-1
bta-miR-1343-5p	COLQ	-0.54	bta-miR-3613b	KPNA6	-1
bta-miR-1343-5p	JUP	-0.54	bta-miR-3613b	USP38	-1
bta-miR-1343-5p	LEPREL4	-0.54	bta-miR-3613b	MRRF	-1
bta-miR-1343-5p	NTN3	-0.54	bta-miR-3613b	CDK12	-1
bta-miR-1343-5p	DCAKD	-0.54	bta-miR-3613b	GAS7	-1
bta-miR-1343-5p	C1orf134	-0.53	bta-miR-3613b	TMBIM6	-1
bta-miR-1343-5p	ELOVL2	-0.53	bta-miR-3613b	USP42	-1
bta-miR-1343-5p	PRKCG	-0.53	bta-miR-3613b	KDELR2	-1
bta-miR-1343-5p	FOXM1	-0.53	bta-miR-3613b	ZFP37	-1
bta-miR-1343-5p	RPH3A	-0.53	bta-miR-3613b	CCDC132	-1
bta-miR-1343-5p	TMEM151B	-0.53	bta-miR-3613b	ZIC5	-1
bta-miR-1343-5p	R3HCC1	-0.53	bta-miR-3613b	SNX30	-1
bta-miR-1343-5p	VPS53	-0.53	bta-miR-3613b	SIK2	-1
bta-miR-1343-5p	CCDC149	-0.53	bta-miR-3613b	IKZF4	-1
bta-miR-1343-5p	LDLRAD2	-0.53	bta-miR-3613b	ARHGAP35	-1
bta-miR-1343-5p	GABBR2	-0.53	bta-miR-3613b	FAM126A	-1
bta-miR-1343-5p	RHBDF2	-0.53	bta-miR-3613b	GOLT1B	-1
bta-miR-1343-5p	CX3CL1	-0.52	bta-miR-3613b	CEP128	-1
bta-miR-1343-5p	LBH	-0.52	bta-miR-3613b	GLE1	-1
bta-miR-1343-5p	ICAM1	-0.52	bta-miR-3613b	AGAP2	-1
bta-miR-1343-5p	ABHD17A	-0.52	bta-miR-3613b	RBM25	-1
bta-miR-1343-5p	TBC1D16	-0.52	bta-miR-3613b	LGI2	-1
bta-miR-1343-5p	GDF11	-0.52	bta-miR-3613b	GAPVD1	-1
bta-miR-1343-5p	CNOT3	-0.52	bta-miR-3613b	UBR2	-1
bta-miR-1343-5p	GINS2	-0.52	bta-miR-3613b	CPD	-1
bta-miR-1343-5p	ANGPTL6	-0.52	bta-miR-3613b	DEDD	-1
bta-miR-1343-5p	FBXO31	-0.52	bta-miR-3613b	CELF2	-0.92
			bta-miR-3613b	FGFBP2	-0.54

Table S3. Predicted mRNA targets of miRNAs differentially expressed between blastocyst SG SM vs. blastocyst FG SM.

miRNAs	Genes	Cumulative Context Score	miRNAs	Genes	Cumulative Context Score
bta-miR-2296	AK2	-0.59	bta-miR-760-5p	TMEM169	-0.54
bta-miR-2296	AP001579.1	-0.57	bta-miR-760-5p	TWIST1	-0.53
bta-miR-2296	ASB16	-0.57	bta-miR-760-5p	WFDC2	-0.61
bta-miR-2296	BARHL2	-0.56	bta-miR-760-5p	WNT9B	-0.5
bta-miR-2296	BBS7	-0.54	bta-miR-760-5p	ZFP36	-0.7
bta-miR-2296	C3orf65	-0.54	bta-miR-760-5p	ZNF3	-0.67
bta-miR-2296	C7orf13	-0.53	bta-miR-17-5p	GPR6	-0.89
bta-miR-2296	C7orf76	-0.79	bta-miR-17-5p	PDCD1LG2	-0.88
bta-miR-2296	C9orf24	-0.74	bta-miR-17-5p	CCL1	-0.79

bta-miR-2296	C9orf78	-0.55	bta-miR-17-5p	PKD2	-0.73
bta-miR-2296	CCDC77	-0.5	bta-miR-17-5p	HAUS8	-0.69
bta-miR-2296	CD226	-0.64	bta-miR-17-5p	PTHLH	-0.64
bta-miR-2296	CHSY3	-0.51	bta-miR-17-5p	IRF9	-0.63
bta-miR-2296	CLDN22	-0.86	bta-miR-17-5p	DERL2	-0.6
bta-miR-2296	CYP3A43	-0.51	bta-miR-17-5p	RHOC	-0.57
bta-miR-2296	DEFB127	-0.76	bta-miR-17-5p	PLEKHA3	-0.53
bta-miR-2296	DEPDC4	-0.63	bta-miR-17-5p	C7orf43	-0.53
bta-miR-2296	EFCAB10	-0.56	bta-miR-17-5p	IL6ST	-0.52
bta-miR-2296	ERCC8	-0.66	bta-miR-17-5p	AGFG2	-0.52
bta-miR-2296	FABP4	-0.66	bta-miR-17-5p	SEMA4B	-0.51
bta-miR-2296	GPA33	-0.6	bta-miR-17-5p	RASL11B	-0.5
bta-miR-2296	HDGFRP3	-0.64	bta-miR-23a	ZNF655	-1.15
bta-miR-2296	KANSL1L	-0.63	bta-miR-23a	ACVR1C	-0.92
bta-miR-2296	KCNC2	-0.57	bta-miR-23a	TFRC	-0.77
bta-miR-2296	KCNIP4	-0.51	bta-miR-23a	PNRC2	-0.76
bta-miR-2296	KIFC1	-0.57	bta-miR-23a	PKP4	-0.75
bta-miR-2296	LST1	-0.66	bta-miR-23a	SNRPC	-0.7
bta-miR-2296	OSBPL10	-0.55	bta-miR-23a	PDE4B	-0.65
bta-miR-2296	PAX4	-0.52	bta-miR-23a	PFDN6	-0.62
bta-miR-2296	PCED1B	-0.74	bta-miR-23a	ZNF23	-0.59
bta-miR-2296	PIM2	-0.76	bta-miR-23a	YES1	-0.59
bta-miR-2296	PRAMEF18	-0.6	bta-miR-23a	TOP1	-0.59
bta-miR-2296	PTMA	-0.6	bta-miR-23a	CCDC82	-0.58
bta-miR-2296	RP11-181C3.1	-0.51	bta-miR-23a	TFPI2	-0.58
bta-miR-2296	RP11-404P21.8	-0.5	bta-miR-23a	DHFR	-0.58
bta-miR-2296	RP11-422N16.3	-0.76	bta-miR-23a	PNMA1	-0.56
bta-miR-2296	SKP1	-0.57	bta-miR-23a	LRAT	-0.56
bta-miR-2296	TCF7L2	-0.69	bta-miR-23a	RALYL	-0.56
bta-miR-2296	TDRD10	-0.81	bta-miR-23a	PDE7A	-0.55
bta-miR-2296	TIGD3	-0.52	bta-miR-23a	C10orf118	-0.54
bta-miR-2296	TMEM51	-0.78	bta-miR-23a	SETD8	-0.54
bta-miR-2296	TMEM66	-0.53	bta-miR-23a	IGSF8	-0.53
bta-miR-2296	TSNAX	-0.5	bta-miR-23a	PKIA	-0.53
bta-miR-2296	UNC45A	-0.61	bta-miR-23a	B3GNT1	-0.53
bta-miR-2296	WNT2	-0.69	bta-miR-23a	MYL12B	-0.53
bta-miR-2296	ZNF791	-0.95	bta-miR-23a	HMGB2	-0.51
bta-miR-450b	B2M	-0.53	bta-miR-23a	SATB1	-0.51
bta-miR-450b	C15orf32	-0.54	bta-miR-23a	ANKHD1	-0.5
bta-miR-450b	C2orf74	-0.67	bta-miR-23b-3p	SS18L2	-1.09
bta-miR-450b	CAMK2N1	-1.01	bta-miR-23b-3p	ELF5	-0.81
bta-miR-450b	CDKN2C	-0.57	bta-miR-23b-3p	ERBB2IP	-0.59

bta-miR-450b	CHMP2B	-0.66	bta-miR-23b-3p	WBP2	-0.55
bta-miR-450b	CT62	-0.62	bta-miR-23b-3p	PPM1D	-0.54
bta-miR-450b	PARK7	-0.55	bta-miR-23b-3p	AUH	-0.53
bta-miR-450b	RAB40A	-0.64	bta-miR-23b-3p	SLC1A1	-0.52
bta-miR-450b	RAET1L	-0.62	bta-miR-23b-3p	ZIC5	-0.5
bta-miR-450b	RP11-169F17.1	-0.67	bta-miR-23b-3p	RAB39B	-0.5
bta-miR-450b	SLC17A6	-0.55	bta-miR-24-3p	STRADB	-0.85
bta-miR-450b	SPTSSB	-0.6	bta-miR-24-3p	TCF7	-0.84
bta-miR-450b	TFRC	-0.53	bta-miR-24-3p	C12orf43	-0.84
bta-miR-450b	TRO	-0.51	bta-miR-24-3p	ENTPD6	-0.84
bta-miR-450b	UBD	-0.59	bta-miR-24-3p	LSM10	-0.79
bta-miR-450b	XRCC4	-0.63	bta-miR-24-3p	SNN	-0.78
bta-miR-450b	ZNF23	-0.57	bta-miR-24-3p	GBA2	-0.76
bta-miR-6535	AC012215.1	-0.51	bta-miR-24-3p	SLCO2B1	-0.75
bta-miR-6535	AC093802.1	-0.81	bta-miR-24-3p	BCL2L11	-0.74
bta-miR-6535	ACOT8	-0.7	bta-miR-24-3p	FAM78B	-0.72
bta-miR-6535	ADRA2A	-0.83	bta-miR-24-3p	DNAJB12	-0.67
bta-miR-6535	AGPAT3	-0.55	bta-miR-24-3p	TSPAN14	-0.67
bta-miR-6535	AL117190.3	-0.54	bta-miR-24-3p	LMBR1L	-0.67
bta-miR-6535	ALKBH5	-0.52	bta-miR-24-3p	KCNK2	-0.66
bta-miR-6535	AMOT	-0.55	bta-miR-24-3p	FASLG	-0.65
bta-miR-6535	AMOTL2	-0.62	bta-miR-24-3p	FST	-0.65
bta-miR-6535	ANKRD52	-0.94	bta-miR-24-3p	ALAD	-0.62
bta-miR-6535	AP1M2	-0.85	bta-miR-24-3p	RAB3IL1	-0.61
bta-miR-6535	APLN	-0.75	bta-miR-24-3p	NEFM	-0.61
bta-miR-6535	APOC4	-0.65	bta-miR-24-3p	CCDC58	-0.61
bta-miR-6535	ARF3	-0.96	bta-miR-24-3p	RAP1B	-0.6
bta-miR-6535	ARL2	-0.58	bta-miR-24-3p	WNT8B	-0.59
bta-miR-6535	ARNT2	-0.52	bta-miR-24-3p	SCML1	-0.59
bta-miR-6535	ARRB1	-0.67	bta-miR-24-3p	MIDN	-0.59
bta-miR-6535	ASNA1	-0.59	bta-miR-24-3p	ABCB9	-0.59
bta-miR-6535	ATCAY	-0.57	bta-miR-24-3p	VAMP5	-0.58
bta-miR-6535	ATP6V0A1	-0.55	bta-miR-24-3p	ZNF697	-0.58
bta-miR-6535	ATP6V0E2	-0.81	bta-miR-24-3p	UBALD2	-0.57
bta-miR-6535	ATXN7L3	-0.9	bta-miR-24-3p	SIRPA	-0.57
bta-miR-6535	BAIAP2L2	-0.55	bta-miR-24-3p	IFNG	-0.56
bta-miR-6535	BARHL1	-0.59	bta-miR-24-3p	BBC3	-0.56
bta-miR-6535	BCAT2	-0.71	bta-miR-24-3p	C10orf62	-0.56
bta-miR-6535	BCL2L2	-0.81	bta-miR-24-3p	ATG4A	-0.56
bta-miR-6535	BEST3	-0.58	bta-miR-24-3p	LIMD2	-0.54
bta-miR-6535	C10orf111	-0.69	bta-miR-24-3p	HNF1B	-0.53
bta-miR-6535	C11orf68	-0.53	bta-miR-24-3p	AC005003.1	-0.53

bta-miR-6535	C19orf35	-0.68	bta-miR-24-3p	SLC25A39	-0.53
bta-miR-6535	C1orf134	-0.53	bta-miR-24-3p	TRIM55	-0.53
bta-miR-6535	C1orf172	-0.57	bta-miR-24-3p	RAP1A	-0.53
bta-miR-6535	C1QL1	-0.52	bta-miR-24-3p	TOP1	-0.52
bta-miR-6535	C20orf27	-0.54	bta-miR-24-3p	CDKN1B	-0.52
bta-miR-6535	C20orf96	-0.64	bta-miR-24-3p	CLLU1	-0.52
bta-miR-6535	C21orf67	-0.59	bta-miR-24-3p	KCTD21	-0.52
bta-miR-6535	C2orf91	-0.78	bta-miR-24-3p	RNF138	-0.52
bta-miR-6535	CACNB1	-0.69	bta-miR-24-3p	FAM45A	-0.51
bta-miR-6535	CACNG7	-0.95	bta-miR-24-3p	CAMK2B	-0.51
bta-miR-6535	CALCOCO1	-0.78	bta-miR-24-3p	B3GNT5	-0.51
bta-miR-6535	CALM2	-0.69	bta-miR-24-3p	RNF115	-0.51
bta-miR-6535	CAMK2A	-0.55	bta-miR-24-3p	C8orf58	-0.51
bta-miR-6535	CD300LB	-0.64	bta-miR-24-3p	TSC22D2	-0.5
bta-miR-6535	CD300LG	-0.55	bta-miR-24-3p	STC2	-0.5
bta-miR-6535	CDCA3	-0.72	bta-miR-24-3p	C16orf59	-0.5
bta-miR-6535	CDKN1A	-0.6	bta-miR-24-3p	SNTB1	-0.5
bta-miR-6535	CHCHD5	-0.51	bta-miR-24-3p	RAB4B	-0.5
bta-miR-6535	CHRNA7	-0.51	bta-miR-2402	C1orf134	-0.98
bta-miR-6535	CHST1	-0.52	bta-miR-2402	ZMAT3	-0.87
bta-miR-6535	CHST3	-0.57	bta-miR-2402	GDI2	-0.86
bta-miR-6535	CLEC3A	-0.54	bta-miR-2402	MRPL32	-0.84
bta-miR-6535	CLSTN3	-0.52	bta-miR-2402	AC117834.1	-0.81
bta-miR-6535	CNN1	-0.54	bta-miR-2402	C19orf53	-0.77
bta-miR-6535	CNTFR	-0.51	bta-miR-2402	GABRA2	-0.74
bta-miR-6535	CNTROB	-0.56	bta-miR-2402	UBE2V2	-0.73
bta-miR-6535	CPLX2	-0.51	bta-miR-2402	AC069547.1	-0.69
bta-miR-6535	CRLF1	-0.68	bta-miR-2402	TMEM239	-0.65
bta-miR-6535	CSDC2	-0.75	bta-miR-2402	PLN	-0.64
bta-miR-6535	CST9	-0.51	bta-miR-2402	LYPLA1	-0.62
bta-miR-6535	CTNNBIP1	-0.55	bta-miR-2402	TMEM182	-0.62
bta-miR-6535	CTNND1	-0.54	bta-miR-2402	MPPED2	-0.6
bta-miR-6535	CTXN1	-0.56	bta-miR-2402	KCMF1	-0.59
bta-miR-6535	CXorf24	-0.59	bta-miR-2402	PRIM2	-0.59
bta-miR-6535	DAGLA	-0.59	bta-miR-2402	SFRP5	-0.59
bta-miR-6535	DCX	-0.55	bta-miR-2402	ALOX15	-0.59
bta-miR-6535	DCXR	-0.5	bta-miR-2402	CLRN3	-0.58
bta-miR-6535	DDAH1	-0.61	bta-miR-2402	FLI1	-0.58
bta-miR-6535	DIRAS2	-0.65	bta-miR-2402	LDOC1L	-0.57
bta-miR-6535	DKFZP761J1410	-0.75	bta-miR-2402	RINT1	-0.56
bta-miR-6535	DPF2	-0.59	bta-miR-2402	MORC1	-0.54
bta-miR-6535	DPP10	-0.73	bta-miR-2402	IL33	-0.54

bta-miR-6535	DTX3	-0.85	bta-miR-2402	CYTH3	-0.54
bta-miR-6535	EFNB1	-1.01	bta-miR-2402	CGNL1	-0.53
bta-miR-6535	EFS	-0.51	bta-miR-2402	PLP1	-0.53
bta-miR-6535	EGR3	-0.58	bta-miR-2402	OLA1	-0.53
bta-miR-6535	ELAVL3	-0.8	bta-miR-2402	CYP2C18	-0.53
bta-miR-6535	ELN	-0.94	bta-miR-2402	FBXO38	-0.52
bta-miR-6535	ELOVL4	-0.53	bta-miR-2402	UHMK1	-0.52
bta-miR-6535	EPHA8	-0.59	bta-miR-2402	PPM1A	-0.52
bta-miR-6535	ETV1	-0.52	bta-miR-2402	GALNT3	-0.52
bta-miR-6535	ETV6	-0.83	bta-miR-2402	PAPPA-AS1	-0.52
bta-miR-6535	FAM83F	-0.61	bta-miR-2402	KLC4	-0.51
bta-miR-6535	FBRS	-0.88	bta-miR-2402	BNIP2	-0.51
bta-miR-6535	FBXL19	-0.56	bta-miR-2402	FAM64A	-0.51
bta-miR-6535	FGF14	-0.69	bta-miR-2402	PLD6	-0.51
bta-miR-6535	FOSB	-0.99	bta-miR-2402	NPY2R	-0.51
bta-miR-6535	FOXL2	-0.54	bta-miR-2402	SLAMF8	-0.5
bta-miR-6535	FOXN2	-0.53	bta-miR-2402	RP11-192H23.4	-0.5
bta-miR-6535	FXYD4	-0.53	bta-miR-2402	TAF13	-0.5
bta-miR-6535	FXYD7	-0.6	bta-miR-2898	PXK	-1.53
bta-miR-6535	FZD4	-0.72	bta-miR-2898	CAPN6	-1.25
bta-miR-6535	GAL3ST1	-0.82	bta-miR-2898	C21orf67	-1.22
bta-miR-6535	GAS8	-0.95	bta-miR-2898	CTF1	-1.15
bta-miR-6535	GATS	-0.75	bta-miR-2898	IGF2R	-1.1
bta-miR-6535	GDF5	-0.52	bta-miR-2898	C15orf32	-1.09
bta-miR-6535	GIGYF1	-0.61	bta-miR-2898	SNURF	-1.04
bta-miR-6535	GLO1	-0.69	bta-miR-2898	PRR18	-1.04
bta-miR-6535	GLYR1	-0.63	bta-miR-2898	PRB3	-1.02
bta-miR-6535	GPATCH2L	-0.58	bta-miR-2898	KRTAP1-3	-1
bta-miR-6535	GPHA2	-0.72	bta-miR-2898	C17orf72	-1
bta-miR-6535	GPR17	-0.53	bta-miR-2898	GPR173	-0.99
bta-miR-6535	HM13	-0.88	bta-miR-2898	KRTAP21-2	-0.98
bta-miR-6535	HMGA1	-0.81	bta-miR-2898	CABP2	-0.93
bta-miR-6535	HOXB1	-0.83	bta-miR-2898	UBE2L3	-0.9
bta-miR-6535	HOXB3	-1.21	bta-miR-2898	TMEM30C	-0.86
bta-miR-6535	HOXC4	-0.63	bta-miR-2898	AGFG2	-0.85
bta-miR-6535	HVCN1	-0.68	bta-miR-2898	CBLN3	-0.83
bta-miR-6535	IER3	-0.61	bta-miR-2898	ONECUT3	-0.82
bta-miR-6535	IGDCC4	-0.54	bta-miR-2898	LELP1	-0.8
bta-miR-6535	IGF2	-0.65	bta-miR-2898	PHLDA2	-0.79
bta-miR-6535	IL1RN	-0.98	bta-miR-2898	SYNDIG1L	-0.78
bta-miR-6535	IL2RG	-0.8	bta-miR-2898	TXNL4A	-0.76
bta-miR-6535	ITFG2	-0.5	bta-miR-2898	CCL11	-0.76

bta-miR-6535	ITIH5	-0.5	bta-miR-2898	CCDC86	-0.75
bta-miR-6535	ITPKB	-0.79	bta-miR-2898	ASCL4	-0.73
bta-miR-6535	JMJD7-PLA2G4B	-0.63	bta-miR-2898	C6orf25	-0.73
bta-miR-6535	JPH4	-0.86	bta-miR-2898	KRTAP4-8	-0.73
bta-miR-6535	KCNAB2	-0.61	bta-miR-2898	COMMD7	-0.72
bta-miR-6535	KCNC1	-0.5	bta-miR-2898	RP11-3B7.1	-0.72
bta-miR-6535	KCND3	-0.5	bta-miR-2898	PRSS37	-0.72
bta-miR-6535	KCNK3	-0.56	bta-miR-2898	FKBP9	-0.72
bta-miR-6535	KIAA0040	-0.72	bta-miR-2898	PIANP	-0.71
bta-miR-6535	KIAA0391	-0.57	bta-miR-2898	GOLGA7B	-0.71
bta-miR-6535	KIF21B	-1.13	bta-miR-2898	ZNF488	-0.71
bta-miR-6535	KLF12	-0.75	bta-miR-2898	NICN1	-0.7
bta-miR-6535	KLF16	-0.57	bta-miR-2898	IGFL1	-0.7
bta-miR-6535	KLK6	-0.67	bta-miR-2898	SHISA7	-0.69
bta-miR-6535	KMT2D	-0.64	bta-miR-2898	TMEM213	-0.69
bta-miR-6535	KRT16	-0.54	bta-miR-2898	RGS8	-0.69
bta-miR-6535	KSR2	-1.23	bta-miR-2898	NRSN2	-0.68
bta-miR-6535	LAMTOR1	-0.6	bta-miR-2898	GPX3	-0.68
bta-miR-6535	LASP1	-0.99	bta-miR-2898	DBNDD2	-0.66
bta-miR-6535	LAT	-0.75	bta-miR-2898	C11orf82	-0.66
bta-miR-6535	LCE2B	-0.53	bta-miR-2898	CDK6	-0.66
bta-miR-6535	LCE2D	-0.9	bta-miR-2898	MRPS18B	-0.65
bta-miR-6535	LEFTY2	-0.59	bta-miR-2898	PABPC1L	-0.65
bta-miR-6535	LEPROT	-0.74	bta-miR-2898	PRR24	-0.65
bta-miR-6535	LHB	-0.54	bta-miR-2898	SCGN	-0.64
bta-miR-6535	LIF	-0.5	bta-miR-2898	NDUFAF2	-0.64
bta-miR-6535	LIMD2	-0.66	bta-miR-2898	ZBED3	-0.64
bta-miR-6535	LMTK3	-0.5	bta-miR-2898	GAPDH	-0.64
bta-miR-6535	LRFN1	-0.61	bta-miR-2898	AL161915.1	-0.64
bta-miR-6535	LY6E	-0.66	bta-miR-2898	AKR1B15	-0.63
bta-miR-6535	MAP2K7	-0.91	bta-miR-2898	DKKL1	-0.63
bta-miR-6535	MARCKSL1	-0.5	bta-miR-2898	PCGF2	-0.62
bta-miR-6535	MARK2	-0.54	bta-miR-2898	SIX2	-0.62
bta-miR-6535	MAX	-0.59	bta-miR-2898	MRAP	-0.62
bta-miR-6535	MAZ	-0.61	bta-miR-2898	AKR1B10	-0.62
bta-miR-6535	MDGA1	-0.62	bta-miR-2898	TEX35	-0.61
bta-miR-6535	MED30	-0.5	bta-miR-2898	MYF5	-0.61
bta-miR-6535	METTL3	-0.53	bta-miR-2898	MOGAT2	-0.61
bta-miR-6535	MFAP2	-0.5	bta-miR-2898	DRAXIN	-0.61
bta-miR-6535	MLN	-0.53	bta-miR-2898	RBFOX3	-0.61
bta-miR-6535	MN1	-0.9	bta-miR-2898	BSND	-0.61
bta-miR-6535	MON1A	-0.57	bta-miR-2898	BCL2L1	-0.6

bta-miR-6535	MSI1	-0.88	bta-miR-2898	SLC8A2	-0.6
bta-miR-6535	MVB12B	-0.53	bta-miR-2898	C5orf51	-0.6
bta-miR-6535	NAB2	-0.69	bta-miR-2898	CTPS2	-0.6
bta-miR-6535	NCR3	-0.86	bta-miR-2898	RSPO4	-0.6
bta-miR-6535	NDP	-0.57	bta-miR-2898	PROK1	-0.59
bta-miR-6535	NEUROD2	-0.53	bta-miR-2898	SH2D4B	-0.59
bta-miR-6535	NFAM1	-0.53	bta-miR-2898	KHDRBS3	-0.59
bta-miR-6535	NFATC2	-0.56	bta-miR-2898	HSPB8	-0.59
bta-miR-6535	NFIC	-0.84	bta-miR-2898	TUBB4A	-0.58
bta-miR-6535	NOTCH3	-0.73	bta-miR-2898	AC026310.1	-0.58
bta-miR-6535	NOVA2	-0.93	bta-miR-2898	SHISA6	-0.58
bta-miR-6535	NPTX1	-0.68	bta-miR-2898	FAM193B	-0.58
bta-miR-6535	NPTXR	-0.88	bta-miR-2898	TMEM178B	-0.58
bta-miR-6535	NRG1	-0.7	bta-miR-2898	HOXC12	-0.58
bta-miR-6535	NXPH2	-0.67	bta-miR-2898	SLC10A3	-0.57
bta-miR-6535	ONECUT3	-0.87	bta-miR-2898	DLX6	-0.57
bta-miR-6535	OST4	-0.5	bta-miR-2898	AC019171.1	-0.57
bta-miR-6535	OXT	-0.56	bta-miR-2898	CALN1	-0.57
bta-miR-6535	PACS1	-0.56	bta-miR-2898	S100A14	-0.56
bta-miR-6535	PACSIN1	-0.58	bta-miR-2898	SCN3B	-0.56
bta-miR-6535	PAFAH1B2	-0.61	bta-miR-2898	VAMP2	-0.56
bta-miR-6535	PCBP4	-0.54	bta-miR-2898	HDGFRP3	-0.56
bta-miR-6535	PCDH1	-1.02	bta-miR-2898	TLCD1	-0.56
bta-miR-6535	PCSK2	-0.72	bta-miR-2898	CLPS	-0.56
bta-miR-6535	PHF1	-0.54	bta-miR-2898	SASH3	-0.56
bta-miR-6535	PIANP	-0.72	bta-miR-2898	ISM1	-0.56
bta-miR-6535	PLA2G6	-0.7	bta-miR-2898	C15orf57	-0.55
bta-miR-6535	PNMA6A	-0.63	bta-miR-2898	DAB2IP	-0.55
bta-miR-6535	POU2F2	-0.55	bta-miR-2898	RAB5C	-0.55
bta-miR-6535	POU3F1	-0.6	bta-miR-2898	PURA	-0.55
bta-miR-6535	PPARD	-0.56	bta-miR-2898	SNAPC3	-0.55
bta-miR-6535	PPDPF	-0.77	bta-miR-2898	IQCF3	-0.55
bta-miR-6535	PPP1R18	-0.87	bta-miR-2898	SRF	-0.55
bta-miR-6535	PRKAG1	-0.5	bta-miR-2898	MC5R	-0.54
bta-miR-6535	PRKCB	-0.56	bta-miR-2898	ZNF213	-0.54
bta-miR-6535	PTGER4	-0.67	bta-miR-2898	CD247	-0.54
bta-miR-6535	PVRL1	-0.92	bta-miR-2898	TBC1D14	-0.54
bta-miR-6535	RAB11FIP5	-0.63	bta-miR-2898	FUK	-0.54
bta-miR-6535	RAB15	-0.61	bta-miR-2898	XXcos-LUCA11.5	-0.54
bta-miR-6535	RAB35	-0.57	bta-miR-2898	ACADS	-0.54
bta-miR-6535	RAD54B	-0.57	bta-miR-2898	SNTA1	-0.54
bta-miR-6535	RARG	-0.58	bta-miR-2898	TBL1X	-0.54

bta-miR-6535	RASL10B	-0.61	bta-miR-2898	CCDC25	-0.54
bta-miR-6535	REEP2	-0.65	bta-miR-2898	DEFB132	-0.54
bta-miR-6535	RHOB	-0.67	bta-miR-2898	FAM155B	-0.53
bta-miR-6535	RHOG	-0.73	bta-miR-2898	TSPAN18	-0.53
bta-miR-6535	RNF141	-0.58	bta-miR-2898	EFNA3	-0.52
bta-miR-6535	RNF39	-0.51	bta-miR-2898	HSPB6	-0.52
bta-miR-6535	ROGDI	-0.87	bta-miR-2898	ARMC10	-0.52
bta-miR-6535	RP11-111M22.2	-0.52	bta-miR-2898	CIR1	-0.52
bta-miR-6535	RP11-159G9.5	-0.63	bta-miR-2898	HMOX1	-0.52
bta-miR-6535	RP11-247C2.2	-0.57	bta-miR-2898	SARS	-0.52
bta-miR-6535	RP6-24A23.6	-1.19	bta-miR-2898	AC005609.1	-0.52
bta-miR-6535	RPS15	-0.52	bta-miR-2898	EIF3L	-0.52
bta-miR-6535	RPS6KA4	-0.83	bta-miR-2898	ACTN1	-0.52
bta-miR-6535	RPS9	-0.75	bta-miR-2898	LDOC1	-0.52
bta-miR-6535	RTBDN	-0.77	bta-miR-2898	PCDH11X	-0.52
bta-miR-6535	RUNX3	-0.92	bta-miR-2898	NAPA	-0.52
bta-miR-6535	SAMD10	-0.68	bta-miR-2898	PHYHIP	-0.51
bta-miR-6535	SAMD12	-0.5	bta-miR-2898	PPM1N	-0.51
bta-miR-6535	SBK1	-0.9	bta-miR-2898	PYDC1	-0.51
bta-miR-6535	SCAMP5	-0.66	bta-miR-2898	TNS1	-0.51
bta-miR-6535	SCGN	-0.72	bta-miR-2898	GBX2	-0.51
bta-miR-6535	SCRT2	-0.9	bta-miR-2898	PTMA	-0.51
bta-miR-6535	SEPN1	-0.51	bta-miR-2898	MARCH9	-0.51
bta-miR-6535	SEPT6	-0.53	bta-miR-2898	AKR1E2	-0.51
bta-miR-6535	SF3B3	-0.54	bta-miR-2898	RANBP10	-0.51
bta-miR-6535	SHISA6	-0.55	bta-miR-2898	LHCGR	-0.51
bta-miR-6535	SLC11A1	-0.63	bta-miR-2898	CNTD1	-0.5
bta-miR-6535	SLC25A23	-0.84	bta-miR-2898	CLPSL1	-0.5
bta-miR-6535	SLC31A2	-0.65	bta-miR-2898	NTM	-0.5
bta-miR-6535	SLC39A13	-0.58	bta-miR-320a	ST7-OT4	-0.73
bta-miR-6535	SLC7A1	-0.51	bta-miR-320a	IFT27	-0.72
bta-miR-6535	SLC7A8	-0.64	bta-miR-320a	PCDHA1	-0.6
bta-miR-6535	SLFN13	-0.56	bta-miR-320a	PBX3	-0.55
bta-miR-6535	SMG6	-0.71	bta-miR-320a	ST8SIA4	-0.55
bta-miR-6535	SNX33	-0.57	bta-miR-320a	VKORC1L1	-0.54
bta-miR-6535	SOCS3	-0.65	bta-miR-320a	TFRC	-0.52
bta-miR-6535	SOX12	-0.81	bta-miR-320a	RP11-160N1.10	-0.5
bta-miR-6535	SP2	-0.52	bta-miR-320a	GTPBP8	-0.5
bta-miR-6535	SRF	-0.73	bta-miR-320a	CTPS1	-0.5
bta-miR-6535	ST3GAL5	-0.55	bta-miR-615	SOD3	-1.28
bta-miR-6535	STAT3	-0.56	bta-miR-615	HSPB7	-1.19
bta-miR-6535	STK35	-1.31	bta-miR-615	C4orf6	-1.15

bta-miR-6535	STX1B	-0.51	bta-miR-615	SPRED3	-1.12
bta-miR-6535	STXBP1	-0.54	bta-miR-615	SLC25A23	-1.05
bta-miR-6535	SYNGAP1	-1.1	bta-miR-615	FOXP3	-1.04
bta-miR-6535	SYNGR1	-1.14	bta-miR-615	SRRM4	-0.99
bta-miR-6535	SYNGR4	-0.67	bta-miR-615	ASTN2	-0.94
bta-miR-6535	SYT5	-0.52	bta-miR-615	CERS1	-0.9
bta-miR-6535	TAF6	-0.7	bta-miR-615	DERL3	-0.86
bta-miR-6535	TBKBP1	-0.51	bta-miR-615	SHMT1	-0.86
bta-miR-6535	TBR1	-0.58	bta-miR-615	GIPC3	-0.86
bta-miR-6535	TCL1A	-0.64	bta-miR-615	TSSK6	-0.85
bta-miR-6535	THRA	-1.37	bta-miR-615	DGCR2	-0.81
bta-miR-6535	TMEM109	-0.5	bta-miR-615	LST1	-0.81
bta-miR-6535	TMEM127	-0.53	bta-miR-615	UPK3A	-0.81
bta-miR-6535	TMEM132E	-0.56	bta-miR-615	CTB-54O9.9	-0.8
bta-miR-6535	TMEM184B	-0.51	bta-miR-615	LRRK1	-0.79
bta-miR-6535	TMEM229B	-1.4	bta-miR-615	RP11-429E11.3	-0.78
bta-miR-6535	TMEM59L	-0.64	bta-miR-615	PARP14	-0.78
bta-miR-6535	TMEM95	-0.54	bta-miR-615	CENPB	-0.78
bta-miR-6535	TNFSF12	-0.58	bta-miR-615	CD3E	-0.76
bta-miR-6535	TP53INP2	-0.57	bta-miR-615	CDH4	-0.76
bta-miR-6535	TPBGL	-0.8	bta-miR-615	AC016722.1	-0.76
bta-miR-6535	TPPP3	-0.5	bta-miR-615	ADM2	-0.76
bta-miR-6535	TRIM46	-0.6	bta-miR-615	RPE	-0.75
bta-miR-6535	TRIM7	-0.7	bta-miR-615	HSF1	-0.74
bta-miR-6535	TSPAN11	-0.77	bta-miR-615	LENEP	-0.74
bta-miR-6535	TSPAN18	-0.74	bta-miR-615	NEDD8	-0.73
bta-miR-6535	TSPYL2	-0.88	bta-miR-615	IKBKB	-0.72
bta-miR-6535	U2AF2	-0.6	bta-miR-615	HSD11B2	-0.72
bta-miR-6535	UBA1	-0.55	bta-miR-615	CTD-2207O23.12	-0.71
bta-miR-6535	UBALD1	-0.76	bta-miR-615	DMPK	-0.71
bta-miR-6535	UBL5	-0.52	bta-miR-615	ENPP7	-0.71
bta-miR-6535	UBTF	-0.72	bta-miR-615	CD19	-0.7
bta-miR-6535	UNC119B	-0.53	bta-miR-615	CTDSP1	-0.7
bta-miR-6535	UNC13A	-1.17	bta-miR-615	ANKRD63	-0.7
bta-miR-6535	VAMP1	-0.72	bta-miR-615	FGFBP2	-0.69
bta-miR-6535	VPS52	-0.66	bta-miR-615	AL138847.1	-0.68
bta-miR-6535	VPS72	-0.74	bta-miR-615	ELP5	-0.68
bta-miR-6535	VSTM2L	-0.69	bta-miR-615	RHOC	-0.67
bta-miR-6535	VSX2	-0.69	bta-miR-615	CTC1	-0.67
bta-miR-6535	VTI1A	-0.53	bta-miR-615	BEAN1	-0.67
bta-miR-6535	WNT5A	-0.66	bta-miR-615	PRKAR1B	-0.67
bta-miR-6535	WWOX	-0.57	bta-miR-615	LTB	-0.67

bta-miR-6535	XKR7	-0.77	bta-miR-615	LILRB3	-0.67
bta-miR-6535	YBX2	-0.53	bta-miR-615	LAMA5	-0.67
bta-miR-6535	ZBTB16	-0.56	bta-miR-615	RERG	-0.66
bta-miR-6535	ZBTB37	-0.67	bta-miR-615	HINT2	-0.66
bta-miR-6535	ZBTB7A	-1.48	bta-miR-615	AC004899.1	-0.66
bta-miR-6535	ZC4H2	-0.66	bta-miR-615	P2RX6	-0.66
bta-miR-6535	ZDHHC6	-0.56	bta-miR-615	EDA	-0.66
bta-miR-6535	ZFP91	-0.54	bta-miR-615	MAZ	-0.66
bta-miR-6535	ZFR	-0.57	bta-miR-615	ALDH16A1	-0.66
bta-miR-6535	ZNF30	-0.55	bta-miR-615	URAD	-0.66
bta-miR-6535	ZNF385A	-1.06	bta-miR-615	BHLHA15	-0.66
bta-miR-6535	ZNF395	-0.57	bta-miR-615	MSLN	-0.66
bta-miR-6535	ZNF488	-0.53	bta-miR-615	PACSIN1	-0.66
bta-miR-6535	ZNF512	-0.77	bta-miR-615	ARIH2	-0.64
bta-miR-6535	ZNF557	-0.58	bta-miR-615	CLIP3	-0.64
bta-miR-6535	ZNF558	-0.63	bta-miR-615	FAM83A	-0.64
bta-miR-6535	ZSWIM1	-0.72	bta-miR-615	CHST13	-0.64
bta-miR-760-5p	AC004899.1	-0.73	bta-miR-615	PARP6	-0.64
bta-miR-760-5p	AC016722.1	-0.99	bta-miR-615	SNAI1	-0.63
bta-miR-760-5p	ACR	-0.57	bta-miR-615	SYNGR1	-0.62
bta-miR-760-5p	AL450307.1	-0.54	bta-miR-615	STX1A	-0.62
bta-miR-760-5p	APH1A	-0.67	bta-miR-615	RAB24	-0.62
bta-miR-760-5p	ARFRP1	-0.56	bta-miR-615	SYNGR2	-0.61
bta-miR-760-5p	ARHGEF2	-0.5	bta-miR-615	ZNF410	-0.61
bta-miR-760-5p	ASIC4	-0.5	bta-miR-615	ATCAY	-0.61
bta-miR-760-5p	BCL2L1	-0.5	bta-miR-615	ZBTB47	-0.61
bta-miR-760-5p	BCL2L2	-0.56	bta-miR-615	TMEM119	-0.61
bta-miR-760-5p	C12orf5	-0.56	bta-miR-615	CDC16	-0.6
bta-miR-760-5p	C14orf132	-0.66	bta-miR-615	KCTD15	-0.6
bta-miR-760-5p	C16orf91	-0.56	bta-miR-615	IFITM5	-0.6
bta-miR-760-5p	C17orf64	-0.52	bta-miR-615	TCF19	-0.6
bta-miR-760-5p	CACNG8	-0.54	bta-miR-615	ZDHHC24	-0.59
bta-miR-760-5p	CD300LB	-1.29	bta-miR-615	KCNQ3	-0.59
bta-miR-760-5p	CDK6	-0.54	bta-miR-615	MEIS2	-0.59
bta-miR-760-5p	CHP2	-0.56	bta-miR-615	PAPPA-AS1	-0.58
bta-miR-760-5p	CT62	-0.98	bta-miR-615	ITPKB	-0.58
bta-miR-760-5p	CYHR1	-0.75	bta-miR-615	ECE1	-0.58
bta-miR-760-5p	DAPP1	-0.52	bta-miR-615	C7orf50	-0.58
bta-miR-760-5p	DDIT4	-0.67	bta-miR-615	PDXP	-0.58
bta-miR-760-5p	DLK2	-0.55	bta-miR-615	PODXL2	-0.58
bta-miR-760-5p	DMKN	-0.78	bta-miR-615	FAM189A1	-0.58
bta-miR-760-5p	DNAJC15	-0.77	bta-miR-615	DDR1	-0.58

bta-miR-760-5p	DOCK1	-0.82	bta-miR-615	ONECUT3	-0.57
bta-miR-760-5p	ELAVL4	-0.67	bta-miR-615	LILRA6	-0.57
bta-miR-760-5p	ENDOV	-0.53	bta-miR-615	ECHDC3	-0.57
bta-miR-760-5p	ESM1	-0.59	bta-miR-615	ZDHHC22	-0.57
bta-miR-760-5p	FAM109A	-0.66	bta-miR-615	RGMA	-0.57
bta-miR-760-5p	GDE1	-0.57	bta-miR-615	MAP2K3	-0.56
bta-miR-760-5p	GEMIN2	-0.63	bta-miR-615	TNFRSF18	-0.56
bta-miR-760-5p	GIMAP6	-0.53	bta-miR-615	SPATA8	-0.56
bta-miR-760-5p	GLIPR2	-0.93	bta-miR-615	ANKRD35	-0.56
bta-miR-760-5p	GORASP1	-1.27	bta-miR-615	FIZ1	-0.56
bta-miR-760-5p	GOT1	-0.55	bta-miR-615	DND1	-0.56
bta-miR-760-5p	HPDL	-0.57	bta-miR-615	ALPP	-0.56
bta-miR-760-5p	ICAM2	-0.58	bta-miR-615	KY	-0.56
bta-miR-760-5p	ILF2	-0.58	bta-miR-615	FAM43B	-0.56
bta-miR-760-5p	JPH2	-0.52	bta-miR-615	INSRR	-0.56
bta-miR-760-5p	KCTD11	-0.72	bta-miR-615	GTF3C5	-0.55
bta-miR-760-5p	KDM1A	-0.5	bta-miR-615	PRR24	-0.55
bta-miR-760-5p	KRTAP9-7	-0.65	bta-miR-615	ASPHD2	-0.55
bta-miR-760-5p	LRP11	-0.76	bta-miR-615	SSTR5	-0.55
bta-miR-760-5p	LTA	-0.68	bta-miR-615	PRR18	-0.55
bta-miR-760-5p	MLANA	-0.51	bta-miR-615	ARMC5	-0.55
bta-miR-760-5p	MRPS18B	-0.53	bta-miR-615	C9orf141	-0.55
bta-miR-760-5p	NAT9	-0.58	bta-miR-615	FBXL12	-0.54
bta-miR-760-5p	OR10A4	-0.53	bta-miR-615	C17orf103	-0.54
bta-miR-760-5p	OR1L3	-0.84	bta-miR-615	SGCA	-0.54
bta-miR-760-5p	OR4M2	-0.5	bta-miR-615	COLQ	-0.54
bta-miR-760-5p	OR5AU1	-0.85	bta-miR-615	HHLA2	-0.54
bta-miR-760-5p	PAX8	-0.72	bta-miR-615	C10orf82	-0.54
bta-miR-760-5p	PCBD2	-0.55	bta-miR-615	EDN3	-0.54
bta-miR-760-5p	PNKD	-0.84	bta-miR-615	DRAXIN	-0.54
bta-miR-760-5p	POLR2J2	-0.73	bta-miR-615	NECAB3	-0.54
bta-miR-760-5p	PON2	-0.64	bta-miR-615	NCAM1	-0.54
bta-miR-760-5p	POSTN	-0.75	bta-miR-615	NKD1	-0.53
bta-miR-760-5p	PRNT	-0.5	bta-miR-615	CYP3A5	-0.53
bta-miR-760-5p	PRPH2	-0.81	bta-miR-615	CAPN15	-0.53
bta-miR-760-5p	RAD54B	-0.54	bta-miR-615	WNT10B	-0.53
bta-miR-760-5p	RASSF2	-0.5	bta-miR-615	C10orf55	-0.52
bta-miR-760-5p	RHOU	-0.87	bta-miR-615	SH3PXD2A	-0.52
bta-miR-760-5p	RP11-144F15.1	-0.56	bta-miR-615	ASPA	-0.52
bta-miR-760-5p	RPGRIP1	-0.67	bta-miR-615	FAM105A	-0.52
bta-miR-760-5p	SEC13	-0.64	bta-miR-615	VKORC1	-0.52
bta-miR-760-5p	SH2D2A	-0.51	bta-miR-615	ADAM12	-0.51

bta-miR-760-5p	SLC25A45	-0.74	bta-miR-615	KREMEN2	-0.51
bta-miR-760-5p	SLC25A48	-0.62	bta-miR-615	PRICKLE2	-0.51
bta-miR-760-5p	SLC26A10	-0.71	bta-miR-615	PRR15L	-0.51
bta-miR-760-5p	SLC29A2	-0.58	bta-miR-615	CCDC155	-0.51
bta-miR-760-5p	SLC4A11	-0.9	bta-miR-615	C2orf48	-0.51
bta-miR-760-5p	STMN4	-0.52	bta-miR-615	CDC14A	-0.51
bta-miR-760-5p	SYP	-0.57	bta-miR-615	TPM3	-0.5
bta-miR-760-5p	TBC1D10C	-0.63	bta-miR-615	TMEM217	-0.5
bta-miR-760-5p	TMED4	-0.73	bta-miR-615	FGD5	-0.5
